# The Transpeptidase Sortase A Binds Nucleic Acids and Mediates Mammalian Cell Labeling

**DOI:** 10.1002/advs.202305605

**Published:** 2024-04-05

**Authors:** Yingzheng Liu, Zhike Lu, Panfeng Wu, Zhaohui Liang, Zhenxing Yu, Ke Ni, Lijia Ma

**Affiliations:** ^1^ College of Life Sciences Zhejiang University Hangzhou 310058 China; ^2^ Westlake Laboratory of Life Sciences and Biomedicine 18 Shilongshan Road Hangzhou 310024 China; ^3^ School of Life Sciences Westlake University 600 Dunyu Road Hangzhou 310030 China; ^4^ Institute of Biology Westlake Institute for Advanced Study 18 Shilongshan Road Hangzhou 310024 China; ^5^ AIdit Therapeutics 1 Yunmeng Road, Building 1 Hangzhou 310024 China

**Keywords:** cell labeling by nucleic acids, GAG, multiplexed scRNA‐seq, sortase

## Abstract

Wild‐type sortase A is an important virulence factor displaying a diverse array of proteins on the surface of bacteria. This protein display relies on the transpeptidase activity of sortase A, which is widely engineered to allow protein ligation and protein engineering based on the interaction between sortase A and peptides. Here an unknown interaction is found between sortase A from *Staphylococcus aureus* and nucleic acids, in which exogenously expressed engineered sortase A binds oligonucleotides in vitro and is independent of its canonical transpeptidase activity. When incubated with mammalian cells, engineered sortase A further mediates oligonucleotide labeling to the cell surface, where sortase A attaches itself and is part of the labeled moiety. The labeling reaction can also be mediated by many classes of wild‐type sortases as well. Cell surface GAG appears involved in sortase‐mediated oligonucleotide cell labeling, as demonstrated by CRISPR screening. This interaction property is utilized to develop a technique called CellID to facilitate sample multiplexing for scRNA‐seq and shows the potential of using sortases to label cells with diverse oligonucleotides. Together, the binding between sortase A and nucleic acids opens a new avenue to understanding the virulence of wild‐type sortases and exploring the application of sortases in biotechnology.

## Graphical abstract

Wild‐type sortase A is an important virulence factor displaying a diverse array of proteins on the surface of bacteria and is engineered to allow protein ligation. Here, authors discover a previously unknown interaction between multiple classes of wild‐type sortases and engineered sortase A and oligonucleotides. This property enables sortase‐mediated oligonucleotide mammalian cell labeling, which can be applied in multiplexing samples for single‐cell sequencing.

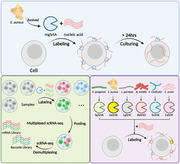

## 1 Introduction

Although sortase A (SrtA) is not essential for bacterial viability, it has attracted broad interest because it functions to display a diverse array of proteins on the bacterial surfaces.^[^ ^1^ ^]^ These displayed surface proteins interact with the immediate bacterial environment and participate in essential bacterial physiological and pathological processes, for example, biofilm formation and the mediation of host cell entry.^[^ ^2^ ^]^ Thus, sortase A is an important virulence factor and is conserved in gram‐positive bacteria. The protein display function of sortase A relies on its cysteine transpeptidase activity that proceeds in a two‐step reaction. Sortase A first attacks the threonine‐glycine bond in an LPXTG motif in the first substrate to form a thioester intermediate, which is further covalently anchored to a cell wall protein with N‐terminal oligoglycine, for example, pentaglycine.^[^ ^1^, ^3^ ^]^

Since the discovery of *Staphylococcus aureus* sortase A and the characterization of its transpeptidase activity, this protein has been engineered for use as a tool in protein engineering, for example, for site‐specific protein modification and protein ligation.^[^ ^4^ ^]^ Through directed evolution or rational design, a variety of SrtA mutants have been developed for enhanced enzymatic activities or a broadened range of substrates. For example, using yeast display, eSrtA was developed with a 140‐fold increased LPETG‐coupling activity compared to wild‐type (WT) SrtA.^[^ ^5^ ^]^ Chen and colleagues developed a FRET‐based platform and induced the evolution of a SrtA mutant with improved enzymatic kinetics.^[^ ^6^ ^]^ Later, the same group reported the construction of mgSrtA, another SrtA mutant capable of the promiscuous labeling of proteins with N‐terminal monoglycine, which facilitates highly efficient peptide labeling on the mammalian cell surface because of its greater range of cellular substrates.^[^ ^7^ ^]^

As a virulence factor and a tool enzyme for protein engineering, SrtA has attracted wide attention in microbiology,^[^ ^8^ ^]^ cell biology,^[^ ^9^ ^]^ and synthetic biology.^[^ ^10^ ^]^ Our understanding of its intrinsic transpeptidase activity has been continuously growing, and its applications in protein engineering have also been extensively explored.^[^ ^11^ ^]^ Indeed, all these studies and applications originated from the interactions between peptides and sortases. Here, we report a previously unknown interaction between nucleic acids and a variety of wild‐type and engineered SrtA. This interaction appears independent of the catalytic residues that are essential to the transpeptidase activity of SrtA. We demonstrated that exogenously expressed SrtA binds to nucleic acids in vitro and mediates the oligonucleotide labeling to the surface of a wide range of mammalian cells in a process that involves cell surface GAGs (glycosaminoglycans). We also found that *Staphylococcus aureus*, a gram‐positive bacteria, from which we retrieved the wild‐type sortase A (WTSrtA) sequence, binds environmental nucleic acids, although direct interaction between nucleic acids and endogenous SrtA requires further characterization. We utilized this interaction property and developed a technique called CellID to facilitate sample multiplexing for scRNA‐seq and showed the potential of using sortases to label cells with diverse nucleotide sequences. Together, this study demonstrated an unrevealed interaction between nucleic acids and sortases, which prompts our understanding of sortases’ physiological function as a virulence factor and their potential application in biotechnology beyond the context of transpeptidation.

## 2 Results

### 2.1 mgSrtA Can Label Mammalian Cells with Oligonucleotide

mgSrtA is one of the engineered SrtA, which was developed to facilitate broader substrates in sortase‐mediated transpeptidation reactions.^[^ ^7^ ^]^ Surprisingly, we found that mgSrtA could mediate the labeling of oligonucleotide to the surface of mammalian cells, which is an unknown property of SrtA as a transpeptidase. As oligonucleotideis highly programmable and easy to analyze, an easy‐to‐use and stable oligonucleotide cell labeling technique would enable various analyses on cells with distinct identifiers.

**Figure 1:**
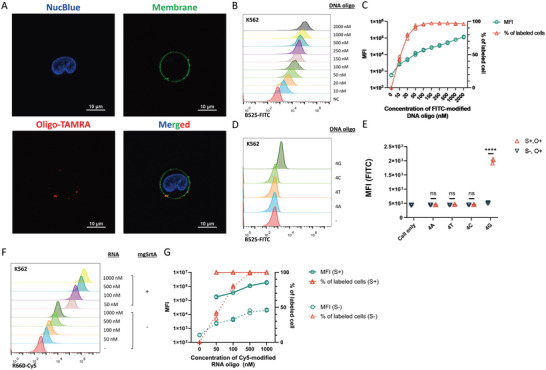
Mammalian cells can be labeled with oligonucleotides in a process mediated by mgSrtA. A) The oligonucleotides localized at the surface of K562 cells after mgSrtA‐mediated cell labeling. Scale bar, 10 µm. Blue, nucleus. Green, membrane. Red, oligonucleotides. B) Flow cytometry quantification of the K562 cells labeled with FITC‐modified DNA oligonucleotides. C) Summary plot of MFIs (mean fluorescence intensities) and percentages of K562 cells labeled with FITC‐modified DNA oligonucleotides at different concentrations. The experiment was repeated 3 times with similar results. D) Flow cytometry quantification of K562 cells labeled with FITC‐modified AAAA (4A), TTTT (4T), CCCC (4C), and GGGG (4G) DNA oligonucleotides. E) Summary plot of K562 cells labeled with FITC‐modified 4‐mer DNA oligonucleotides (S: mgSrtA; O: oligonucleotide). Data are represented as mean ± SD of three replicates. F) Flow cytometry quantification of K562 cells labeled with Cy5‐modified RNA oligonucleotides. G) Summary plot of K562 cells labeled with Cy5‐modified RNA oligonucleotides at different concentrations. MFI, mean fluorescence intensity. Data are represented as the mean of three replicates. ns: not significant, ^****^ *p* < 0.0001 by unpaired 2‐tailed *t*‐test. A, D, E, 0.5 million K562 cells were incubated with 20 µm mgSrtA and 100 nm oligonucleotides at 37 °C for 10 min. B, C, F, G, 0.5 million K562 cells were incubated with 20 µm mgSrtA at 37 °C for 5 min and incubated for another 10 min in the presence of oligonucleotides.

To better understand the properties of mgSrtA‐mediated oligonucleotide cell labeling, we conducted a series of cell labeling experiments to examine the stability of the labeled oligonucleotide, the nucleotide preference, and the labeling efficiencies of various cell types. First, we performed mgSrtA‐mediated cell labeling with modified fluorescent DNA oligonucleotides by incubating K562 cells, mgSrtA, and DNA oligonucleotides for 10 min at 37 °C. The labeled cells were observed under confocal microscopy and then subjected to quantitative analysis by flow cytometry (Figure S1, Supporting Information). Cells were labeled with oligonucleotides only when incubated with mgSrtA and oligonucleotides at the same time. To further detect the location of the labeled oligonucleotide on cells by mgSrtA, we stained the cell and found fluorescent signals located on the cell membrane (**Figure** **1**A; Figure S2, Supporting Information). Then, we titrated the oligonucleotide concentration for optimal labeling efficiency and noticed that almost all cells were labeled when 100 nm DNA oligonucleotides were applied in both K562 and Jurkat cells. The mean fluorescence intensity (MFI) was positively correlated with the oligonucleotide concentration, and did not reach a plateau when even 2000 nm oligonucleotide was applied (Figure 1B,C; Figure S3, Supporting Information). Furthermore, we found that the fluorescent signals of the positively labeled cells were detectable after 24 h under standard cell culture conditions regardless of the different modifications of oligonucleotides (Figure S4A,B, Supporting Information). Both confocal and flow cytometry data demonstrated that mgSrtA mediates a nucleotide cell labeling phenomenon, in which the labeling process is easy, and the signal is stable under regular cell culture conditions.

We also conducted mgSrtA‐mediated cell labeling on a variety of laboratory cultured cell lines (Figure S10A–C, Supporting Information) and multiple primary cell types (Figure S10D–F, Supporting Information), which were all successfully labeled by DNA oligonucleotides, although the labeling efficiencies varied across cell types. The broad range of successfully labeled cell types suggested that mgSrtA‐mediated oligonucleotide cell labeling was generalizable among mammalian cells.

### 2.2 mgSrtA‐Mediated Cell Labeling Shows Nucleotide Selectivity

Next, we asked whether applying different DNA oligonucleotides would reveal a nucleotide preference for mgSrtA‐mediated cell labeling. We synthesized four types of 4‐mer DNA oligonucleotides, each composed of only one type of nucleotide. FITC‐modified 4A (AAAA), 4T (TTTT), 4C (TTTT), and 4G (GGGG) DNA oligonucleotides were used to label cells, and the cells were then subjected to flow cytometry analysis. Each type of 4‐mer DNA oligonucleotide was detected on cells, as demonstrated by the increase in MFI compared to that of the non‐mgSrtA‐treated control cells (Figure 1D,E). Among the four DNA oligonucleotides, the 4G oligonucleotide produced the highest MFI, indicating that mgSrtA‐mediated oligonucleotide cell labeling exhibits a preference for guanine. We repeated the cell labeling with 4‐mer DNA oligonucleotides modified with biotin and TAMRA, and the results indicated that this preference is independent of modification (Figure S5, Supporting Information).

The guanine preference was further confirmed by an unbiased sequence screening (Figure S6A, Supporting Information), in which a DNA oligonucleotide embedded with a PCR handle, a random sequence (12‐nt), and a poly(A) tail was screened for its cell labeling ability in the presence (mgSrtA+) or absence (mgSrtA‐) of mgSrtA. We reasoned that the sequence composition of the 12‐nt random nucleotide differs from the ability of the DNA nucleotide for cell labeling. Thus, we captured the DNA oligonucleotides labeled on cells by SMART‐seq through the poly(A) tail and enriched the products of oligonucleotides by PCR according to the PCR handle before library preparation. We found that the random 12‐nt nucleotide of that DNA oligonucleotide from the positively labeled cells shows sequence conservation of continuous guanine bases (Figure S6B, Supporting Information). Moreover, by conducting cell labeling and flow cytometry analysis, we also demonstrated that both single‐stranded (ss) DNA and double‐stranded (ds) DNA could label cells mediated by mgSrtA (Figure S7, Supporting Information). Together, these results suggested that nucleic acids could be effectively and stably attached to the surface of K562 cells in the presence of mgSrtA. The preference for guanine nucleotides over the others implied a selectivity of mgSrtA‐mediated cell labeling.

Next, we examined whether other forms of nucleic acids could be labeled on cell surfaces mediated by mgSrtA. We labeled K562 and Jurkat cells with Cy5‐modified RNA oligonucleotides at different oligonucleotide concentrations. Similar to the DNA oligonucleotides, the RNA oligonucleotides were labeled to both cell lines in a mgSrtA‐dependent manner, and the labeling efficiencies were positively correlated with the concentrations of the RNA oligonucleotides (Figure 1F,G; Figure S8, Supporting Information). Additionally, a biotin‐modified peptide nucleic acid (PNA) was used to label K562 cells under the same conditions, yielding a magnitude higher MFI on the positively labeled cells (Figure S9, Supporting Information). Since the backbone of PNA is a peptide bond, this data also suggested that the deoxyribose or ribose sugar backbone of nucleic acids is not required in mgSrtA‐mediated cell labeling.

Overall, we found that mgSrtA mediates the labeling of the mammalian cell surfaces with various forms of nucleic acids, and the fluorescence signals of the oligonucleotides remain detectable after 24 h under standard cell culture conditions, suggesting the great potential of mgSrtA for diverse oligonucleotide cell labeling.

### 2.3 Applying mgSrtA‐Mediated Cell Labeling for Multiplexed scRNA‐seq

The mgSrtA‐mediated robust labeling of oligonucleotides to cell surface encouraged us to explore its application in single‐cell sequencing. When several samples were subjected to scRNA‐seq (single‐cell RNA‐seq) for transcriptome comparison, sample multiplexing would eliminate batch effects and reduce cost compared to processing each sample individually. Moreover, sample multiplexing is sometimes required as the sample amounts may not satisfy the optimal cell number for starting. Thus, we reasoned that mgSrtA could be used to record the identities of cells by labeling them with oligonucleotides before mixing multiple samples into one tube for single‐cell encapsulation. These oligonucleotide labels could be further resolved by high‐throughput sequencing, and cellular identities could be assigned to transcriptomes from the multiplexed samples. We named this labeling approach CellID to indicate its applications in single‐cell technologies, for example, multiplexed scRNA‐seq.

We investigated a variety of parameters to optimize the cell labeling conditions for CellID. We started with 100 nm DNA oligonucleotide to optimize the reaction buffer (Figure S11, Supporting Information), pH (Figure S12, Supporting Information), and temperature (Figure S13, Supporting Information). We included various conditions compatible with cell‐based assays and concluded that mgSrtA could effectively mediate cell labeling with DNA oligonucleotides at 37 °C in PBS or HBSS between pH 6.5–8.0 (Figure S12, Supporting Information). Commonly used cell culture media were also compatible, with or without fetal bovine serum (FBS), but the labeling efficiencies were lower than those in PBS or HBSS (Figure S11, Supporting Information). Cell labeling also occurred at a lower temperature, for example, 4 °C or room temperature (RT), but the efficiency was slower (Figure S13, Supporting Information). Additionally, we optimized the EDTA concentration for effective termination to make CellID labeling more manageable. The results demonstrated that labeling could be terminated with 30 mm EDTA, and termination was more effective with Ca^2+^‐dependent mgSrtA (Figure S14, Supporting Information).

**Figure 2:**
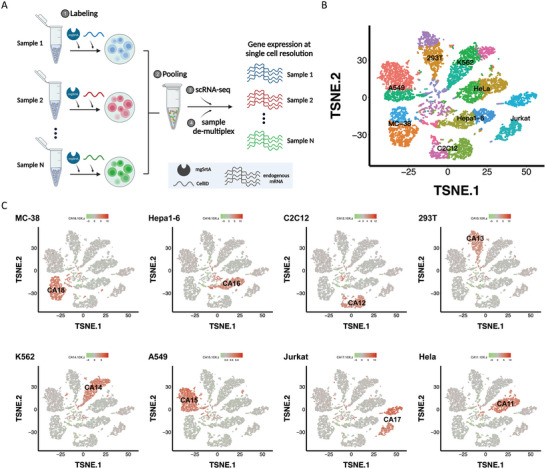
Application of mgSrtA‐mediated cell labeling in multiplexed scRNA‐seq. A) Flowchart of multiplexed scRNA‐seq by CellID. B) Cell‐type annotations according to the transcriptome. *n* = 10392. C) CellID accurately distinguished cells derived from eight samples.

Next, we applied the CellID for cell labeling in multiplexed scRNA‐seq. As a demonstration, we employed mgSrtA and eight kinds of DNA oligonucleotides to label five human cell lines and three mouse cell lines before pooling all the cells for scRNA‐seq in the 10x platform (**Figure** **2**A; Figure S15, Supporting Information). The different species and distinct cell types could be used to annotate individual cells using single‐cell transcriptomes, which were compared to the cellular identities defined by CellID. We reasoned that the CellID assignment of cells should match their assignment from transcriptome annotation according to the expression profiles. We used Seurat^[^ ^12^ ^]^ to generate clusters for 10,392 cells that passed the quality filtering of the 10X standard data processing pipeline and visualized the cells in a tSNE plot. According to the transcriptome annotation, each of the eight cell types was projected to 2–3 tSNE clusters of single cells (Figure 2B). We then assigned CellID to single cells according to the sequenced oligonucleotide, which was labeled to the cell surface before sample multiplexing and represented the cell identity. Strikingly, the CellID assignment perfectly matched the cell identities assigned by transcriptome annotation (Figure 2C). Together, these data showed that CellID could enable simultaneous analysis of multiplexed samples in scRNA‐seq and demonstrated its application as programmable cell barcoding.

### 2.4 mgSrtA Binds with Oligonucleotides

**Figure 3:**
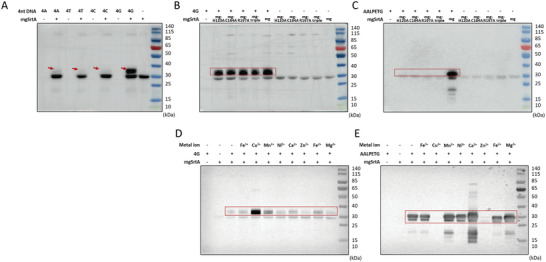
Oligonucleotides bind with mgSrtA in vitro. A) 10 µm mgSrtA incubated with 20 µm 4‐mer DNA oligonucleotide at 37 °C for 30 min (the DNA oligonucleotides were modified by 5′ biotin and 3′ FITC). The red arrow points to the product bands. B) The mgSrtA mutants incubated with 4G DNA oligonucleotide. C) The mgSrtA mutants incubated with 20 µm biotin‐modified AALPETG peptide. mgSrtA‐triple represents the mgSrtA mutant with H120A, C184A, and R197A mutations. D) mgSrtA incubated with 4G DNA oligonucleotide in the presence of 100 µm metal ions. E) mgSrtA incubated with the AALPETG peptide in the presence of 100 µm metal ions. The product band is in the red box. The membranes were incubated with a mouse anti‐biotin primary antibody and a goat anti‐mouse secondary antibody.

Besides exploring the CellID application, we also tried to dissect the mechanism of mgSrtA‐mediated cell labeling. We first examined whether mgSrtA binds oligonucleotides in vitro. We incubated biotin‐modified 4‐mer DNA oligonucleotides with mgSrtA and observed shifted bands by Western blotting (WB) (**Figure** **3**A). Consistent with the nucleotide preference of mgSrtA in cell labeling, the 4G oligonucleotide yielded stronger WB bands than the 4A, 4T, and 4C oligonucleotides, so does in Electrophoretic Mobility Shift Assay (EMSA) (Figure S16, Supporting Information). Applying a series of guanine oligonucleotides (4G, 6G, 8G, 15G, and 20G) generated bands of increasing size, which aligned with the increased length of these guanine oligonucleotides (Figure S17, Supporting Information). These results demonstrated that mgSrtA bound oligonucleotides in vitro in the absence of cell labeling.

The WB results encouraged us to further investigate how the DNA oligonucleotides were bound to mgSrtA. As mgSrtA should have been denatured during WB, the presence of the product bands suggested a strong binding of mgSrtA with the 4G oligonucleotide. However, it remained possible that the band shifts resulted from a strong affinity due to a hydrophobic bond or ionic bond between the 4G oligonucleotide and incompletely denatured mgSrtA, even in a 2% SDS buffer. To rule out the possibility of an affinity‐dependent complex, we pretreated mgSrtA in 2% SDS at 95 °C for 10 min and returned to room temperature before incubating with the 4G oligonucleotide. No product band was detected when the mgSrtA was pretreated with 2% SDS (Figure S18, Supporting Information), which suggested that an affinity for incompletely denatured mgSrtA was insufficient to yield the observed band shifts, and the binding between mgSrtA and the DNA oligonucleotide was not fully rely on the hydrophobic interaction and electrostatic interaction. We also conducted microscale thermophoresis (MST) quantification, and the data showed the K_d_ of the binding between mgSrtA and the oligonucleotide was 5.13 ± 0.87 µm (Figure S19, Supporting Information). Collectively, these findings supported that mgSrtA binds oligonucleotides (with a preference for guanine) strongly.

### 2.5 The Binding Between mgSrtA and Oligonucleotides Is Independent of the Transpeptidase Activity of the Parental Wild‐Type Sortase A

The canonical function of WTSrtA is transpeptidation, by which bacterial proteins with LPXTG sorting motifs are cleaved between threonine and glycine and displayed on the cell wall. To test whether the binding between mgSrtA and DNA oligonucleotides is related to the intrinsic transpeptidase activity of the enzyme, we introduced mutations at residues critical for the transpeptidase activity of WTSrtA.^[^ ^3^ ^]^ These mgSrtA mutants (H120A, C184A, R197A and H120A+C184A+R197A) retained binding to the 4G oligonucleotide but lost transpeptidation activity with the AALPETG peptide,^[^ ^13^ ^]^ which is a substrate of sortase‐catalyzed transpeptidation (Figure 3B,C). We also examined the activities of other mgSrtA mutants. mgSrtA‐mono (N132A+K137A+Y143A), which carries mutations that abolish the dimerization activity of SrtA,^[^ ^14^ ^]^ also bound the 4G oligonucleotide but not the AALPETG peptide (Figure S20, Supporting Information). The WB data of these mgSrtA mutants indicated that the binding between mgSrtA, the engineered SrtA, and the oligonucleotide is independent of the transpeptidation activity of the WTSrtA.

Additionally, inspired by the involvement of Ca^2+^ in sortase‐mediated transpeptidation,^[^ ^15^ ^]^ we screened multiple cations to determine whether any of them strengthened the binding between mgSrtA and DNA oligonucleotides. We added various metal cations at 100 µm to the in vitro reaction of mgSrtA and the 4G oligonucleotide. The addition of Cu^2+^ increased the band intensity of the binding product compared to those in the no‐cation control and in reactions with other metal cations (Figure 3D), suggesting that the binding between mgSrtA and oligonucleotides could be enhanced by Cu^2+^, although the mechanism is unclear. However, the addition of Cu^2+^ almost completely inhibited the production of the binding product of mgSrtA and AALPETG peptide, and the addition of Ca^2+^ increased the binding between mgSrtA and AALPETG as reported (Figure 3E). Together, the binding between mgSrtA and oligonucleotide appears distinct from the formation of a thioacylenzyme intermediate in the transpeptidation reaction catalyzed by the WTSrtA.^[^ ^3^, ^13^, ^16^ ^]^

### 2.6 mgSrtA Bridges Oligonucleotides on the Cell Surface

**Figure 4:**
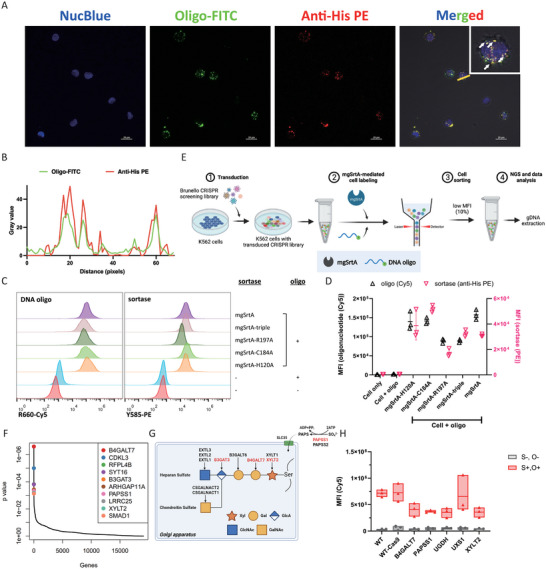
mgSrtA bridges oligonucleotides on the cell surface. A) Confocal images of K562 cells labeled with FITC‐modified oligonucleotides and incubated with anti‐his PE antibody. Blue, nucleus. Green, oligonucleotides. Red, mgSrtA. Yellow, the colocalization of mgSrtA, and the oligonucleotide. The white arrow points to the colocalization sites. Scale bar, 20 µm. B) Fluorescence intensity profiles along the yellow line in the merged image. C) The signals of the labeled oligonucleotide and anchored mgSrtA or its mutants on cells were detected by flow cytometry. D) Summary plot of K562 cells labeled with Cy5‐modified DNA oligonucleotides mediated by mgSrtA and its mutants. MFI, mean fluorescence intensity. Data are represented as mean ± SD of three replicates. E) A schematic flowchart of CRISPR screening to identify the cellular proteins involved in or contributing to mgSrtA‐mediated oligonucleotide cell labeling. F) The top hits of CRISPR screening. Genes are ranked (*x*‐axis) by *p*‐value (*y*‐axis). G) The pathway for biosynthesis of chondroitin sulfate and heparan sulfate. Genes enriched by CRISPR screening are marked in red. H. Validation of the CRISPR screening candidates. Candidate genes were knocked out from K562 cells to establish the engineered K562 cells. We established monoclonal K562 cell lines by knocking out B4GALT7, PAPSS1, UGDH, UXS1, and XYLT2, respectively. A wild‐type (WT) and a Cas9 knock‐in (WT‐Cas9) K562 cells were used as controls. These K562 cells were labeled with Cy5‐modified oligonucleotides, and the fluorescent signals were analyzed by following cytometry. S: mgSrtA; O: oligonucleotide. MFI, mean fluorescence intensity. The experiment was conducted in triplicates.

After characterizing the direct binding between oligonucleotides and mgSrtA, we next investigated how mgSrtA mediated oligonucleotide labeling on the mammalian cell surface. Interestingly, after exposure of the K562 cells to the oligonucleotides and mgSrtA, we detected the signals of mgSrtA, labeled oligonucleotides, and mammalian cells by confocal microscopy and found that mgSrtA colocalized with the oligonucleotides on the surface of the labeled cells (**Figure** **4**A). Additionally, the fluorescence intensities of mgSrtA and the oligonucleotides in the merged image were correlated (Figure 4B). These data indicate that mgSrtA itself is involved in the labeling of the mammalian cell surface with oligonucleotides.

We then used flow cytometry to quantify the signals of the fluorescent oligonucleotides and the attached mgSrtA. In this experiment, we also included multiple mgSrtA mutants known to bind with oligonucleotides in previous WB analyses. Interestingly, the signal of the attached SrtA seems positively correlated with the corresponding signal of fluorescent oligonucleotide in general (Figure 4C,D), which supported the idea that the participation of mgSrtA as part of the labeled moieties on the cell surface. However, an LPXTG peptide could be attached to the cell surface by only mgSrtA but not by mgSrtA‐triple, mgSrtA‐R197A, mgSrtA‐C184A, or mgSrtA‐H120A, which is consistent with the lack of binding between the LPXTG peptide and these mgSrtA mutants (Figure S21, Supporting Information). In summary, these data suggested that the presence of the oligonucleotide signal on the cell surface is mgSrtA dependent, and mgSrtA is required as part of the labeled moiety.

### 2.7 GAGs May Contribute to Attach mgSrtA on the Cell Surface

To further dissect how mgSrtA and mutant forms were attached to the cell surface and to mediate the oligonucleotide labeling on cells, we examined which cell surface components might contribute. Lipids, proteins, and carbohydrates are the three macromolecules composing the mammalian cell membrane. Given that the fluorescence signals of mgSrtA and the labeled oligonucleotides on the cell surface appeared to be aggregated (Figure 4A,B), we focused on proteins and carbohydrates rather than the widely distributed membrane lipids. We used various proteinase and deglycosylase enzymes to disturb the proteins and carbohydrates on the cell surface and monitored the mgSrtA‐mediated cell labeling signals for changes. All proteinases we tested caused a ≈50% or more decrease in labeling signal intensity (Figure S22, Supporting Information), and the promiscuous proteinases trypsin and proteinase K caused ≈75% decrease in the fluorescence signals.

Deglycosylases also decreased the cell labeling efficiency, although the extent varied across enzymes and cell types. We noticed that the combined use of heparinase 1, 2, and 3 and chondroitinase ABC dramatically impacted the labeling efficiency, yielding 50–70% reductions in the fluorescent signals on K562, Jurkat, and 293T cells compared to the HBSS buffer‐only sample (Figure S23, Supporting Information). In contrast, the use of heparinase 1, 2, 3, or chondroitinase ABC alone showed a weaker impact on the labeling efficiency (Figures S24–S25, Supporting Information). Pretreatment with PNGase F and O‐glycosidase did not decrease the labeling efficiency obviously (Figure S26, Supporting Information). Decreased signals were also observed in Jurkat and 293T cells but not in K562 cells upon hyaluronidase digestion (Figure S27, Supporting Information). The reduction in labeling efficiency on cells pretreated with proteinases and deglycosylases led us to hypothesize that glycosaminoglycans (GAGs) might be involved in the attachment of mgSrtA and hence the mediation of cell labeling with oligonucleotides.

To pinpoint the key components on the cell surface that contribute to mgSrtA attachment and oligonucleotide cell labeling, we applied whole‐genome CRISPR screening (Figure 4E). We used the Brunello CRISPR library^[^ ^17^ ^]^ to knock out genes in K562 cells that were then labeled with oligonucleotides. We reasoned that the transduced K562 cells that lost or showed decreased efficiency of mgSrtA‐mediated oligonucleotide cell labeling may represent a genotype, in which genes critical for cell labeling have been knocked out. Under this rationale, the transduced K562 cells that displayed bottom 15% MFIs were collected by fluorescence‐activated cell sorting (FACS) after two rounds of selection for labeling efficiency, and the sgRNA counts of these cells were compared with those of a control group of K562 cells transduced with the same CRISPR library but without any further treatment. Among the top ten hits from the CRISPR screening, *XYLT2* (xylosyltransferase 2) encodes a xylosyltransferase that initiates the formation of a tetrasaccharide linker between GAG and a core protein,^[^ ^18^ ^]^ and *B4GALT7* (beta‐1,4‐galactosyltransferase 7) and *B3GAT3* (beta‐1,3‐glucuronyltrasferase 3) encode two galactosyltransferases responsible for linker elongation.^[^ ^19^ ^]^ *PAPSS1* (3′‐phosphoadenosine 5′‐phosphosulfate synthase 1) encodes one of the two synthases that form PAPS, which is a sulfate donor for GAG sulfation^[^ ^20^ ^]^ (Figure 4F‐G). To verify the screening results, we conducted mgSrtA‐mediated oligonucleotide labeling to K562 cell lines with XYLT2 or B4GALT7 knocked out. Compared to wild‐type K562, we observed 50% and 40% reductions in the signals of the oligonucleotide labeled on XYLT2‐KO K562 and B4GALT7‐KO K562, respectively (Figure 4H). The ability of the knockout cell lines to bind with mgSrtA was also influenced (Figure S28A, Supporting Information). Similar results were obtained for SrtE1‐mediated, SrtE2‐mediated, and SrtF‐mediated cell labeling (Figure S28B,C, Supporting Information).

To further confirm the participation of GAG in the attachment of mgSrtA to the cell surface, we examined whether mgSrtA binds with heparin in vitro and in cell cultures. We examined the binding between biotin‐modified heparin and mgSrtA by WB and observed a binding product when Cu^2+^ was present (Figure S29A,B, Supporting Information). Furthermore, the bands of heparin‐mgSrtA complexes were also observed in EMSA (Figure S29C, Supporting Information). Consistent with the in vitro binding data, heparin could also be labeled to cell surface mediated by mgSrtA (Figure S30, Supporting Information). Together, our data indicated that mgSrtA is attached to the cell surface to mediate oligonucleotide and peptide labeling through GAG, for example, heparin.

### 2.8 Oligonucleotide Binding Is a Previously Unknown Property of Wild‐Type Sortase A

**Figure 5:**
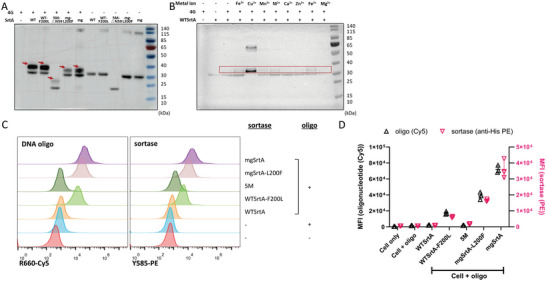
Oligonucleotide binding is a previously unknown characteristic of wild‐type sortase A. A) 10 µm WTSrtA or its mutants incubated with 20 µm 4G DNA oligonucleotide at 37 °C for 30 min (the DNA oligonucleotide was modified by 5′ biotin and 3′ FITC). The red arrow points to the product bands. B) WTSrtA incubated with 4G DNA oligonucleotide in the presence of 100 µm metal ions. The product band is in the red box. Western Blots were incubated with a mouse anti‐biotin primary antibody and a goat anti‐mouse secondary antibody. C) The signals of the labeled oligonucleotide and anchored WTSrtA or its mutants on cells were detected by flow cytometry. D) Summary plot of K562 cells labeled with Cy5‐modified DNA oligonucleotides mediated by WTSrtA and its mutants. Data are represented as mean ± SD of three replicates.

The mgSrtA was engineered from the WTSrtA of *Staphylococcus aureus* to allow more expansive substrates for transpeptidation.^[^ ^7^ ^]^ We investigated whether the ability to bind oligonucleotides and mediate oligonucleotide cell labeling was a previously unrevealed property of WTSrtA or emerged with the protein engineering of SrtA. To answer this question, we expressed and purified WTSrtA and three engineered SrtA (5M,^[^ ^5^ ^]^ mgSrtA‐L200F,^[^ ^6^ ^]^ and mgSrtA^[^ ^7^ ^]^) and quantified their binding with oligonucleotide both in vitro and in cell labeling. The 5Mm was named after five residue (P94R, D160N, D165A, K190E, and K196T) mutated from WTSrtA, mgSrtA‐L200F contains three further mutated residues (D124G, Y187L, and E189R) in addition to the mutations in 5M, and mgSrtA carries additional mutation F200L from mgSrtA‐L200F. Strikingly, both WT and the three engineered SrtA bound oligonucleotides in vitro (**Figure** **5**A), which suggests that binding oligonucleotides is a previously unrevealed property of WTSrtA. We also found that the binding between WTSrtA and oligonucleotides could be enhanced by the metal ion Cu^2+^ (Figure 5B), which is also a property of mgSrtA (Figure 3F).

Next, we applied both the purified WT and the three engineered SrtA to label cells with oligonucleotides and examined the signals of the labeled oligonucleotide and the attached SrtA. Unlike mgSrtA and mgSrtA‐L200F, which carry the most mutated residues, WTSrtA poorly attached to the cell surface and mediated weak cell labeling with oligonucleotides, as did 5M (Figure 5C,D). We further expressed and purified WT‐F200L, a mutant with F200L inserted directly into WTSrtA, and observed increased signals of the attached WT‐F200L and the labeled oligonucleotide (Figure 5C,D). Together, the in vivo binding and the mammalian cell labeling results demonstrated that WTSrtA binds oligonucleotides, whereas mediating oligonucleotide cell labeling is an emergent property of engineered SrtA resulting from directed evolution, to which the mutation F200L seems to contribute.

### 2.9 *S. aureus* Cells Are Labeled with Oligonucleotides on Their Surface

**Figure 6:**
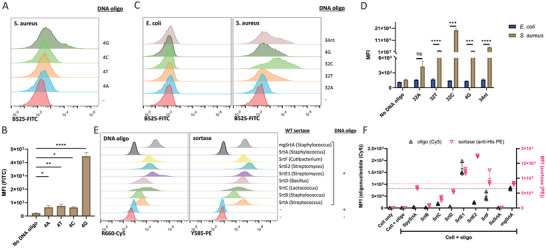
Gram‐positive bacteria can be labeled with oligonucleotides at their surface. A) 2 million *S. aureus* incubated with 1 µm 4‐mer FITC‐modified DNA oligonucleotides at 37 °C for 20 min. B) Summary plot of *S. aureus* labeled with 4‐mer FITC‐modified DNA oligonucleotides. Data are represented as mean ± SD of three replicates. C) 2 million *S. aureus* or *E. coli* incubated with 1 µm FITC‐modified DNA oligonucleotides at 37 °C for 15 min. D) Summary plot of *S. aureus* and *E. coli* oligonucleotide labeling. Data are represented as mean ± SEM of three replicates. E) A variety of wild‐type sortases were used to label K562 cells with oligonucleotides. F) Summary plot of K562 cells labeled with Cy5‐modified DNA oligonucleotides mediated by various WT sortases. The dotted lines represent the level of mgSrtA. MFI, mean fluorescence intensity. Data are represented as mean ± SD of three replicates. ns: not significant, ^*^ *p* < 0.05, ^**^ *p* < 0.01, ^***^ *p* < 0.001, ^****^ *p* < 0.0001 by one‐way ANOVA or unpaired 2‐tailed *t*‐test.

Given the observation that both the mgSrtA and the purified WTSrtA could bind DNA oligonucleotides, we were curious whether the endogenous SrtA on the surface of *S. aureus* could directly bind extracellular DNA oligonucleotides. We incubated the FITC‐modified 4G, 4C, 4T, and 4A DNA oligonucleotides with *S. aureus* as we did for the mammalian cells, except no exogenously expressed and purified enzyme was added. We used flow cytometry to quantify the signals of *S. aureus* and found that the 4G oligonucleotide exhibited a 3‐fold higher signal than the other three DNA oligonucleotides (**Figure** **6**A,B), which was consistent with the pattern of in vitro binding between WTSrtA and oligonucleotides and the mammalian cell labeling.

To further verify that surface SrtA contributed to the labeling of *S. aureus* with DNA oligonucleotides, we repeated the DNA oligonucleotide labeling on *Escherichia coli*, a gram‐negative bacterium with no surface sortase expression. With various DNA oligonucleotides, the fluorescence signals detected from *E. coli* remained at the baseline level of the “no DNA oligonucleotide” control, while signals detected from *S. aureus* were at least one magnitude higher except for that of the 32A oligonucleotide (Figure 6C,D). Among the examined DNA oligonucleotides, the signals of the 32C oligonucleotide were 100‐fold higher than those of the “no DNA oligonucleotide” control. *Escherichia coli* can only be labeled in the presence of exogenous SrtA (Figure S31, Supporting Information). Together, these results demonstrated that gram‐positive bacteria such as *S. aureus* but not gram‐negative bacteria such as *E. coli* could be directly labeled with oligonucleotides on their surface.

Since multiple classes of sortases are expressed on the bacterial surface, we then explored an expanded list of WT sortases that could be employed to enable oligonucleotide labeling on the surface of mammalian cells. We expressed sortase A from *Streptococcus*, sortase B from *Staphylococcus*, sortase C from *Lactococcus*, sortase D from *Bacillus*, sortases E1 and E2 from *Streptomyces*, sortase F from *Cutibacterium* and used these WT sortases to label mammalian cells with oligonucleotides. Signal quantification demonstrated the presence of the attached sortases and the labeled oligonucleotide on the cell surface (Figure 6E,F). The signals of the attached sortases showed that various types of WT sortases from different bacterial strains can bind to the mammalian cell surface, while the sortase A from *S. aureus* showed the weakest signal. Surprisingly, sortase E1 may exhibit a stronger ability than mgSrtA to label the cell surface with oligonucleotides, as supported by the higher MFI of the labeled oligonucleotides. Sortase F, sortase E2, and sortase C all showed signals more than one magnitude higher than that of the no‐sortase control. Noticeably, although the signal of the attached sortase F is the highest among all sortases we tested, the signal of its labeled oligonucleotide was not among the strongest ones. Moreover, we observed a guanine preference in these WT sortases (Figure S32, Supporting Information). Collectively, these data demonstrated that multiple classes of purified wild‐type sortases could attach themselves to the surface of mammalian cells and mediate oligonucleotide cell labeling, implying that these properties may positively contribute to the fitness of gram‐positive bacteria.

## 3 Discussion

SrtA is an important virulence protein of gram‐positive bacteria, as it could display various proteins on the surface of bacteria. This protein display relies on the transpeptidase activity of SrtA, which was later employed to develop tools for protein ligation and protein engineering. Both the physiological function and the application potential of SrtA made it an attractive enzyme in the fields of microbiology, cell biology, and bioengineering. However, no interactions have been found between nucleic acids and endogenous or purified SrtA. In this study, we demonstrated that a variety of wild‐type sortases, including sortases A, B, C, D, E1, E2, and F, and engineered SrtA could bind oligonucleotide in vitro. We also found that many of the wild‐type sortase and the engineered SrtA can attach to the surface of mammalian cells, which further mediates the nucleotide labeling to the cell surface. These findings illustrated unknown interactions between sortases and nucleic acids and between sortases and mammalian cells, which not only shed light on other possible virulent properties of wild‐type sortases but also expanded the toolbox of wild‐type and engineered sortases.

Starting from an unexpected observation, we found that mgSrtA, an engineered SrtA, mediates the labeling of oligonucleotides to the surface of mammalian cells. We then demonstrated that the labeling process is practical and gentle for cultured cells, and the labeled oligonucleotides are stable on the cell surface for at least 24 h. These properties collectively made mgSrtA a great cell labeling tool, in which diverse nucleotide sequences could be used to distinguish cells from a cell population and later be resolved by high‐throughput sequencing. As a demonstration, we applied a multiplexed scRNA‐seq experiment to exemplify the efficiency and specificity of mgSrtA‐mediated oligonucleotide cell labeling (CellID) and exhibited the potential of CellID in identifying cells from a cell population. In addition to labeling cells in vitro, applying CellID in vivo may help to mark cells that participate in an interesting biological process, for example, labeling tumor infiltrated lymphocytes (TILs) by injecting CellID in solid tumors in vivo, or recording immune cell interactions.^[^ ^21^ ^]^

Long‐term monitoring of the oligonucleotide‐labeled cells further exemplified another potential of mgSrtA in delivering nucleic acids into cells. We observed the signals of Cy5‐modified oligonucleotide 120 h post‐cell labeling, both on the cell surface and inside the cells, as demonstrated by flow cytometry and imaging analysis (Figure S4C–F, Supporting Information). Since the extended detection window was only observed on Cy5‐modified oligonucleotide, it is not clear whether Cy5 modification contributed to the extended time of labeling since the electrostatic and hydrophobic interactions may stabilize the binding between Cy5 and the lipid bilayer on the cell membrane.^[^ ^22^ ^]^ To exclude the possible interference from the modifications, we incubated HEK293T cells with the GFP‐expressing plasmid with the presence of mgSrtA and wild‐type sortases and surprisingly observed intracellular fluorescence (Figure S33, Supporting Information), suggesting that mgSrtA and wild‐type sortases could facilitate the expression of exogenous nucleic acids in mammalian cells. Nucleic acid drugs, such as siRNA and anti‐sense oligo (ASO), naturally have difficulties passing through the membrane of human cells due to the negative charges and usually require delivery vectors or modifications to be effectively delivered into cells. The nucleic acid binding activity of sortases may serve as a new delivery tool to deliver this type of drug into cells.

These attractive application potentials encouraged us to explore the underlying mechanism of the mgSrtA‐mediated oligonucleotide cell labeling. After conducting a series of in vitro and cell labeling experiments, we confirmed that mgSrtA binds oligonucleotide in relatively high affinity (5.13±0.87 µm). Docking simulation predicted the possible binding configurations between the oligonucleotide and mgSrtA. The resultant docking model was compared with the crystal structure of the complex of WTSrtA and the LPXTG peptide (PDB ID: 2KID). The simulation indicated that a 4‐mer poly‐guanine could bind to a separate activation site but in the same binding pocket as the peptide (Figure S34, Supporting Information), which allows the oligonucleotide to be accommodated in mgSrtA. However, further evidence such as mass spectrometry analysis and crystallization structure will be needed to illustrate whether the binding of sortase and oligonucleotides is covalent and to identify the modified residues of sortase.

Our data also proved that mgSrtA is part of the labeled moiety when mediating the labeling of the oligonucleotide to the mammalian cell surface. To uncover the cell surface proteins that interact with mgSrtA, we used proteinases and deglycosylases to pre‐treat cells before cell labeling and further conducted whole‐genome CRISPR screenings to identify genes critical for successful cell labeling. Multiple lines of evidence pinpointed that cell surface GAGs were involved in mgSrtA‐mediated oligonucleotide cell labeling. Interestingly, a parallel CRISPR screening on peptide‐labeled cells indicated that GAG also contributed to mgSrtA‐mediated peptide cell labeling (Figure S35, Supporting Information). mgSrtA was initially developed to mediate peptide labeling on an expanded list of substrates on mammalian cells,^[^ ^7^ ^]^ while imaging data from this study further indicates that the labeled peptides on cell surface also co‐localized with mgSrtA (Figures S36–S37, Supporting Information). Considering that sortase A serves as a transpeptidase in the canonical transpeptidation and is not part of the final product of the reaction, the co‐localization of mgSrtA and peptide on cell surface we found in this study suggested there is an alternative or additional mechanism to enable the mgSrtA‐mediated peptide cell labeling.

Besides the engineered mgSrtA, we also asked whether wild‐type sortases could naturally bind oligonucleotide and attach on the surface of mammalian cells or whether these properties actually emerged during the protein engineering to SrtA. Interestingly, although the wild‐type sortase A from *Staphylococcus aureus* (WTSrtA) could barely attach to the surface of mammalian cells (Figure 5C,D), which indicated that the binding to the cell surface is a new property of the engineered mgSrtA, we found that the oligonucleotide‐binding activity is a generic property of WTSrtA (Figure 5A). Moreover, convergently evolved WT sortases of other classes share the oligonucleotide‐binding property (Figure 6E,F), including sortase A from *Streptococcus*, sortase B from *Staphylococcus*, sortase C from *Lactococcus*, sortase D from *Bacillus*, sortases E1 and E2 from *Streptomyces*, and sortase F from *Cutibacterium*. The abovementioned wild‐type sortases also appear to bind to cell surfaces, at least with the presence of oligonucleotides, which may serve as promising parental proteins to be further engineered to mediate oligonucleotide or peptide binding and mammalian cell labeling.

The evolutionary conserved property of wild‐type sortases indicated that the binding between nucleic acids and sortases may be common in gram‐positive bacteria and relevant to their fitness. It is known that sortase A contributes to the formation of biofilm, in which environmental polysaccharides, protein, lipids, and nucleic acids are utilized to build an external film structure to increase bacterial viability, for example, to guard the bacteria against antibiotics.^[^ ^23^ ^]^ Previous reports have also demonstrated that the digestion of extracellular DNA could disrupt biofilm formation.^[^ ^23^, ^24^ ^]^ The DNA‐binding activity of the *Staphylococcus aureus* sortase A (and other classes of sortases) characterized in this study established a connection between the contributions of sortase A and extracellular DNA in bacteria biofilm formation. This connection further emphasized that SrtA is a valuable target to disrupt the formation of biofilms, which usually help bacteria survive antibiotic exposure. Actually, sortase A has already been a popular drug target, and inhibitors have been developed to disrupt its transpeptidase activity for displaying bacterial surface virulence proteins. In the future, with a further understanding of the potential interaction between bacteria sortases and extracellular nucleic acids, the therapeutic potential of sortase A, as well as other wild‐type sortases, may be explored.

In summary, this study expanded our understanding of the function of SrtA beyond its transpeptidase activity in gram‐positive bacteria and its application in protein ligation and protein engineering. The shared property of nucleic acids binding by multiple classes of wild‐type sortases suggests a fitness advantage, which requires further efforts to characterize in an endogenous context. The property of mediating nucleic acids cell labeling allows straightforward and flexible barcoding of living cells in various scenarios, for example, cell multiplexing for single‐cell sequencing. Meanwhile, the transportation of nucleic acid drugs in vivo may also be applied.

## 4 Experimental Section

### Cell Labeling—*Cell Culture*

K562 and Jurkat cells were cultured in RPMI 1640 medium (Sigma R8758) supplemented with 10% FBS and 1% penicillin/streptomycin. 293T, HeLa, A549, MC‐38, Hepa1‐6, and C2C12 cells were cultured in DMEM (Sigma D6429) supplemented with 10% FBS (Gemini 900‐108) and 1% penicillin/streptomycin (Gibco 15140‐122). H1 cells were cultured in mTeSR1 Basal Medium (Stemcell 85 851) with 1× mTeSR1 supplement (Stemcell 85 852). Cells were incubated at 37 °C with 5% CO_2_.

### Cell Labeling—*Preparation of DNA Oligonucleotides, RNA Oligonucleotides and Double‐Stranded DNA*

Oligonucleotides were ordered from General Biol (Anhui, China), GenScript (Nanjing, China), and Genewiz (Suzhou, China). Peptides were synthesized by Scilight Biotechnology (Beijing, China). The sequences of oligonucleotides and peptides are shown in Table S1 (Supporting Information).

The powder form of the Cy5‐modified RNA oligonucleotide was diluted with RNase‐free H_2_O, and aliquots were stored in a −80 °C freezer.

The dsDNA was amplified in a 50 µL PCR, including 1 uL plasmid template, 2.5 µL of 10 µm 5′biotin modified forward primer, 2.5 µL of 10 µm 5′biotin modified reverse primer, 19 µL of nuclease‐free water and 25 µL of Q5 High‐Fidelity 2× Master Mix (NEB M0494). The reaction was performed under the following conditions: 98 °C for 30 s; 30 cycles of 98 °C for 10 s, 67 °C for 30 s, and 72 °C for 10 s; and a final extension step of 72 °C for 2 min. In total, four PCRs were combined, concentrated with an Amicon Ultra 0.5 mL 3 kDa MWCO centrifugal filter (Millipore UFC5030BK), and purified and size‐selected with 2× AMPure XP beads (Beckman A63882). The amplification products were eluted in 40 µL of nuclease‐free H_2_O. dsDNAs were added to 0.5 million K562 cells at a final concentration of 40 nm in the presence of 20 µm mgSrtA, and the cultures were incubated at 37 °C for 10 min.

**S short Bio F1**:

5′‐GTGTTACGGCGTTTCTCCAAC‐3′

**S short Bio R1**:

5′‐CCACCTACTTTGGAGTCGAGG‐3′

**S short Bio F2**:

5′‐GACAGATAGCACCAGGTCAGAC‐3′

**S short Bio R2**:

5′‐GCGTACTGCCTGCTTGGT‐3′

### Cell Labeling—*Sortases Protein Expression and Purification*

The DNA sequences of WT and engineered SrtA and WT sortases from different bacterial strains were cloned into the pET‐28a backbone with an N‐terminal 6xHis tag. The vector was transformed into and expressed in *E. coli* BL21 (DE3). IPTG (0.2 mm) was added to each liter of *E. coli* culture when the OD600 reached 0.6. The cultures continued growing overnight at 18 °C before the bacterial cells were harvested by centrifugation. The cell pellet was resuspended in 40 mL of lysis buffer (20 mm Tris‐HCl, pH 7.8, 500 mm NaCl) supplemented with protease inhibitors. The lysate was sonicated for 4 s followed by 4 s of rest for 150 cycles at 35% vibration amplitude with a one‐half‐inch probe on a Branson SFX550. After sonication, the lysate was centrifuged, and the supernatant was filtered using a 0.45 µm filter (Millipore SLHVR33RB) before being loaded into a gravity column with 2.5 mL of Ni‐NTA Agarose (Qiagen 1 018 244). The column was washed with 20 mL of washing buffer (20 mm Tris‐HCl, pH 7.8, 500 mm NaCl, 40 mm imidazole), and the target protein was eluted with 40 mL of elution buffer (20 mm Tris‐HCl, pH 7.8, 500 mm NaCl and 250 mm imidazole). Amicon Ultra15 centrifugal filters were applied when a small volume was desired. The purified protein was then stored at −80 °C in 10% glycerol as a stock solution. The sequences of sortases are shown in Table S2 (Supporting Information).

### Cell Labeling—*Labeling*

To label cells, the DNA, RNA, or peptide were incubated with 0.5 million cells in the presence of mgSrtA (20 µm) in a 50 µL reaction at 37 °C for 10 min. The typical substrate concentration was 100 nm for DNA and RNA and 20 µm for peptide. Reactions were terminated with 50 mm EDTA.

We have two loading sequences for labeling reactions. In simple mode, 0.5 million cells were incubated with oligonucleotides and 20 µm mgSrtA at the same time at 37 °C for 10 min. But in some cases, higher concentrations of oligonucleotides or less substrate on unfamiliar cells may cause repression of the labeling reaction. In order to make sure that there were adequate mgSrtA attaching cell surface to bride oligonucleotides, the “Enzyme‐1st ” labeling method was adopted.

In the “Enzyme‐1st” labeling method, 0.5 million cells were first incubated with 20 µm mgSrtA at 37 °C for 5 min, followed by the addition of oligonucleotides and incubation at 37 °C for another 10 min. The reaction conditions are detailed in Table S1 (Supporting Information).

### Flow Cytometry Analysis

Before flow cytometry analysis, 0.5 million cells were washed twice in 1 mL of cold PBS (Sigma D8537) supplemented with 1% BSA. If the labeled substrates were modified with biotin, cells need to incubate with 1 µL PE Streptavidin (Biolegend 405 204) or APC Streptavidin (Biolegend 405 207) in 100 µL PBS on ice for 30 min before being analyzed by flow cytometry. To capture the signal of sortases, cells were incubated with 1 uL PE anti‐His tag (Biolegend 362 603) in 100 µL PBS on ice for 30 min. After washing, the cells were resuspended in 200 µL of cold PBS and analyzed on a BC CytoFLEX LX. The analyses were performed by FlowJo V10.

### Screening for Nucleotide Sequences—*Sample Preparation*

After the labeling reaction, the cells were washed three times with PBS. Five hundred cells were counted for both the labeled sample and an unlabeled control sample for SMART‐seq library preparation.

### Screening for Nucleotide Sequences—*SMART‐seq Library Preparation*

The SMART‐seq (TAKARA 634 889) workflow was followed until the purification of amplified cDNA. The products of oligonucleotides were enriched for 2× bead selection and eluted in 12 µL of nuclease‐free H_2_O.

To generate the final library, cDNA in 2 µL of bead elution was amplified in a 50 µL PCR, including 0.5 µL of 10 µm “dT primer”, 0.5 µL of 10 µm “P7 Primer ”, 22 µL of nuclease‐free water and 25 µL of NEBNext Ultra II Q5 Master Mix (NEB M0544). The reaction was performed under the following conditions: 98 °C for 30 s; 10/20 cycles (10 cycles for the labeled sample and 20 cycles for the unlabeled control sample) of 98 °C for 10 s, 53 °C for 30 s and 72 °C for 15 s; and a final extension step of 72 °C for 2 min. In total, five PCRs were combined, concentrated with an Amicon Ultra 0.5 mL 30 kDa MWCO centrifugal filter (Millipore UFC5030BK), and purified and size‐selected with 1.8× AMPure XP beads (Beckman A63882). The amplification products were eluted in 30 µL of nuclease‐free H_2_O.

In the 2nd PCR, 2 µL of template from the 1st PCR was used in each 50 µL reaction, including 25 µL of NEBNext Ultra II Q5 Master Mix (NEB M0544), 0.5 µL of 10 µm “P5 Primer”, 0.5 µL of 10 µm “P7 Primer” and 22 µL of nuclease‐free water. The PCR program was set as follows: 98 °C for 30 s; 8 cycles of 98 °C for 10 s, 66 °C for 30 s, and 72 °C for 20 s; and a final extension step of 72 °C for 2 min. A total of twelve reactions were pooled and concentrated with an Amicon Ultra 0.5 mL 30 kDa MWCO centrifugal filter (Millipore UFC5030BK). The products were purified and size‐selected twice with 1.4× AMPure XP beads.

**dT primer**:

5′‐CTACACGACGCTCTTCCGATCTATGGTGAGCAAGGGCGNNNNNNNNNNTTTTTTTTTTTTTTTTTTTTTTTTTTTTTTVN‐3′

**P5 Primer**:

5′‐AATGATACGGCGACCACCGAGATCTACACTCTTTCCCTACACGACGCTCTTCCG‐3′

**P7 Primer**:

5′‐CAAGCAGAAGACGGCATACGAGATATATCAGTGTGACTGGAGTTCAGACGTGTGC‐3′

### Screening for Nucleotide Sequences—*SMART‐seq Data Processing*

The SMART‐seq NGS data was first removed from adapters and aligned to the pre‐designed oligonucleotide sequence using bowtie2. The nucleotides with the range of the 12‐nt random sequences were further visualized using WebLogo.^[^ ^25^ ^]^

### Imaging

Cells were collected, washed twice with PBS, and then divided into aliquots of 0.5 million cells in 50 µL of HBSS (Sigma H6684) per tube. The cells were labeled with 100 nm oligonucleotide in the presence of 20 µm mgSrtA at 37 °C for 10 min. At the end of incubation, the cells were washed twice with HBSS. To observe the nuclei, cells were resuspended with 1 mL PBS and incubated with two drops of NucBlue Live ReadyProbes (Invitrogen R37605) at RT for 15 min. To observe the cell membrane, cells were incubated with 1 µL CellMask Green PM Stain (Invitrogen C37608) in 1 mL PBS at 4 °C for 10 min. To observe the signal of mgSrtA, cells were incubated with 5 uL PE anti‐His tag (Biolegend 362 603) in 100 µL PBS on ice for 0.5 h. After washing residual dye with PBS at the end of incubation, cells were transferred to Nunc Lab‐Tek Chambered Coverglass (Thermo Scientific 155 411) at a density of 20000 cells in 300 µL of HBSS per well. Confocal images were acquired by Nikon C2Si 60× oil objective lens under the FITC or TAMRA channel, with a laser power = 0.5.

### Western Blotting

DNA oligonucleotides and mgSrtA were mixed and incubated in HBSS at 37 °C for 30 min. At the end of incubation, the reaction was stopped by adding 1× loading dye, and samples were denatured at 95 °C for 15 min. The mixture was then separated by 4–20% Bis‐Tris PAGE (GenScript M00656, M00657) and transferred onto a nitrocellulose membrane (Merck HATF00010). The membranes were blocked by incubating with 5% BSA in 1× TBST (Sangon Biotech C520009) and incubated for 2 h at RT or overnight at 4 °C with an anti‐biotin antibody (Abcam ab201341) at a 1:500 dilution in 5% BSA TBST. Then, the membranes were washed three times with TBST and incubated for 1 h at RT with HRP‐conjugated secondary antibodies (Invitrogen 31 430) at a 1:5000 dilution in 5% BSA TBST. After being washed three times with TBST, the membranes were imaged using SuperSignal West Pico PLUS (Thermo 34 580) by GE AI680RGB.

### MST

The fluorescently labeled target oligo‐Cy5 (purchased from General Biol) was adjusted to 50 nm with reaction buffer (20 mm HEPES, 300 mm NaCl, pH 6.8) supplemented with 0.1% Tween 20. The unlabeled protein‐ligand mgSrtA was dialyzed into the reaction buffer and then diluted to prepare appropriate serial concentration gradients. For each assay, an unlabeled ligand was mixed with an equal volume of fluorescently labeled target and loaded into standard Monolith NT.115 capillaries (NanoTemper Technologies). Measurements were performed at 25 °C with a Monolith NT.115 instrument (NanoTemper Technologies) using 20% excitation power and 40% MST power. The Kd value was calculated using MO. Affinity Analysis software (NanoTemper Technologies) was used to determine the mean ± SEM from four independent experiments with a single site‐specific binding model.

### Enzyme Digestion

Enzyme digestion was performed with 0.5 million cells in a 50 µL reaction. Cells were incubated with proteinase or glycosidase before CellID labeling. A total of 0.5 million cells were counted and treated with glycosidases (Heparinase I NEB P0735S, Heparinase II NEB P0736L, Heparinase III NEB P0737S, Chondroitinase ABC Sigma C3667, PNGase F NEB P0705S, O‐Glycosidase NEB P0733S, Hyaluronidase Sigma 389 561) at 37 °C (except heparinases at 30 °C) for 1h or proteinase (0.25% Trypsin‐EDTA Gibco 25 200 072, TrypLE Express Gibco 12 605 028, Collagenase 1 Gibco 17 100 017, Collagenase 2 Gibco 17 101 015, Collagenase 4 Gibco 17 104 019, Accutase Gibco A1110501, Dispase Stemcell 0 7923, Proteinase K Invitrogen 25 530 031) at 37 °C for 5 min. At the end of the incubation, the cells were pelleted by centrifugation for 3 min at 500 ×g and washed twice with 1 mL of PBS. The cells were then incubated with 20 µm mgSrtA at 37 °C for 5 min in HBSS, followed by the addition of oligonucleotide to a final concentration of 100 nm and incubation at 37 °C for another 10 min.

### CRISPR Screening—*CRISPR Screening Experiments*

The lentivirus Brunello CRISPR screening library was transduced into 120 million K562 cells with stable Cas9 expression, at MOI = 0.3. Seventy‐two hours post‐transduction, 2 µg mL^−1^ puromycin was added to eliminate the nontransduced cells. After seven days, 15 million cells were labeled with 100 nm DNA oligonucleotide (Cy5‐ or FITC‐modified) or 20 µm peptides (FITC‐ or biotin‐modified) in the presence of 20 µm mgSrtA. The cells were washed three times in DPBS before being subjected to cell sorting. In the first round selection, cells with the highest 20% MFI and the lowest 20% MFI were sorted on a BD FACSAria Fusion. Ten days later, 12 million cells derived from the top 20% cells were labeled and cells with the highest 15% MFI of them were collected. Meanwhile, 12 million cells derived from the bottom 20% cells were labeled and cells with the lowest 15% MFI of them were collected. Genomic DNA (gDNA) was extracted from the sorted cells. The gRNA cassette was amplified in a 50 µL PCR, including 600 ng cDNA, 1.25 µL of 10 µm “P5‐Brunello”, 1.25 µL of 10 µm “P7‐Brunello”, 25 µL of NEBNext Ultra II Q5 Master Mix and nuclease‐water to final volume of 50 µL. The reaction was performed under the following conditions: 98 °C for 30 s; 24 cycles of 98 °C for 10 s, 65 °C for 30 s, and 72 °C for 20 s; and a final extension step of 72 °C for 2 min. In total four PCRs were combined, concentrated with an Amicon Ultra 0.5 mL 30 kDa MWCO centrifugal filter, and purified and size‐selected with 1.8× AMPure XP beads. The amplification products were eluted in 30 µL of nuclease‐free H_2_O. A parallel starting reference, without cell labeling and cell sorting, was included as a control sample for CRISPR screening.

**P5‐Brunello**:

5′‐ AATGATACGGCGACCACCGAGATCTACACTATAGCCTACACTCTTTCCCTACACGACGCTCTTCCGATCTTGTGGAAAGGACGAAACAC −3′

**P7‐Brunello**:

5′‐ CAAGCAGAAGACGGCATACGAGATATAGCGTCGTGACTGGAGTTCAGACGTGTGCTCTTCCGATCTCCGACTCGGTGCCACTTTTTCAA −3′

### CRISPR Screening—*CRISPR Screening Data Analysis*

The CRISPR screening NGS libraries were sequenced as 150‐bp paired ends. The sequencing reads were first undergone adapter removal by ‘cut adapter’^[^ ^26^ ^]^ with parameters ‘‐n 1 ‐e 0.11 ‐O 15 ‐m 16’. The sequences were then aligned to the designed CRISPR library sequences using bowtie2 with “–np 0 –n‐ceil L,0,0.2 –very‐sensitive”.^[^ ^27^ ^]^ The read number of each gRNA was counted from the Brunello CRISPR library in both the mgSrtA labeled sample and the control sample (starting reference). The read numbers were then normalized according to the sequencing depth of each NGS library, and an enrichment score was calculated for each gRNA (thus the targeted gene) using MAGeCK.^[^ ^28^ ^]^

### CellID Labeling for Multiplexed scRNA‐seq—*Sample Preparation*

Approximately 0.5 million cells per sample were pelleted by centrifugation at 500 ×g for 3 min. The pellets were washed twice with PBS and resuspended in 50 µL of labeling buffer containing 100 nm oligonucleotide and 20 µm mgSrtA. Cells were incubated in a labeling buffer at 37 °C for 10 min, and then labeling was terminated by the addition of 50 mm EDTA. The cells were then pelleted at 500 ×g for 3 min at 4 °C and washed three times with 1 mL of cold PBS. PBS was supplemented with 1% BSA and 30 mm EDTA in the 1st wash and with 0.04% BSA in the 2nd and 3rd washes. The cells were resuspended in PBS with 0.04% BSA. Multiple samples were then combined in the desired ratio and subjected to 10x Genomics analysis. During sample preparation, each tube was prerinsed with 1 mL of PBS containing 1% BSA. After each round of washing, the supernatant was transferred to a new prerinsed tube.

### CellID Labeling for Multiplexed scRNA‐seq—*scRNA‐seq Library Preparation*

The 10x Genomics Single Cell 3′ v3 workflow was followed until the cDNA amplification step. To amplify the CellID oligonucleotides together with the cDNA, 0.5 µL of 2 µm “Nested PCR primer” was added to the cDNA PCR mix. When CS CellID (with the 10x capture sequence at the 3′ end) was used, another 0.5 µL of 2 µm “Partial Read1N primer” was added.

**Nested PCR primer**: 5′‐CCACTCACATCCACTACCAACACT‐3′.

**Partial Read1N primer**: 5′‐GCAGCGTCAGATGTGTATAAGAGACAG‐3′.

The cDNA amplification products were size‐selected with 0.6× AMPure XP beads. The long fragment fraction was subjected to cDNA library preparation following the manufacturer's instructions.

For the supernatant of the 0.6× bead selection, another 1.4× beads were added to enrich the short fragments originating from the CellID experiment. The beads were washed twice with 200 µL of 80% ethanol and eluted in 40 µL of Buffer EB (Qiagen 1 014 608). The polyA CellID library was amplified using the “P5 Sample index primer” and “P7 Read2 index primer”. PCR was performed in a 50 µL volume including 5 µL of cDNA, 1.25 µL of 10 µm forward primer, 1.25 µL of 10 µm reverse primer, 17.5 µL of nuclease‐free water, and 25 µL of NEBNext Ultra II Q5 Master Mix (NEB M0544). The reactions were carried out as follows: 98 °C for 30 s; 8–16 cycles of 98 °C for 10 s, 55 °C for 30 s, and 72 °C for 15 s; and a final extension step of 72 °C for 2 min. The library was cleaned up with 1.2× beads.

**P5 Sample index primer**:

5′‐AATGATACGGCGACCACCGAGATCTACACTAATCTTAACACTCTTTCCCTACACGACGCTC‐3′.

**P7 Read2 index primer**:

5′‐CAAGCAGAAGACGGCATACGAGATCTATCGCTGTGACTGGAGTTCAGACGTGTGCTCTTCCGATCTTCACATCCACTACCAACACTCT‐3′.

### CellID Labeling for Multiplexed scRNA‐seq—*10x scRNA‐seq Data Analysis*

The sequencing data of the scRNA‐seq libraries were processed by Cell Ranger (provided by 10x Genomics Inc.). Each transcript was first assigned with a 10x Cell Barcode. Transcripts with the same Cell Barcode were considered to originate from the same cell and were used to determine the cell type identity of that cell. For each cell, the sequencing reads aligned to the CellIDs were further counted, which were further normalized as z‐scores. The most dominant CellID, which exhibited the highest z‐score, determines the representative CellID of that cell. Thus, for each cell in the analysis, the classification performance of CellID can be evaluated by the cell type identity.

### Plasmids Labeling

0.5 million HEK‐293T cells were incubated with 25 µm mgSrtA at 37 °C for 10 min in HBSS, followed by the addition of 3 µg plasmids for the total volume of 50 µL and incubated at 37 °C for another 30 min. At the end of incubation, the supernatant of the reaction system was removed and cells were resuspended with 1 mL DMEM completed medium. 200 µL cell suspension was seeded to the 48‐well plate with the addition of another 1 mL DMEM completed medium. Then, cells were grown at 37 °C with 5% CO_2_ for 72 h and imaged with Leica Thunder DMI8.

### EMSA

DNA oligonucleotides and mgSrtA were mixed and incubated in HBSS at 37 °C for 30 min. At the end of incubation, 1 µL 10× loading dye was added to each 10 µL reaction. The mixture was then separated by 6% PAGE (Beyotime GS306S) and transferred onto a nylon membrane (Merck FFN15). After cross‐linked by 254 nm UV, 120mJ cm^−2^ for 90 s, the membranes were blocked by incubating with blocking buffer (Beyotime GS009‐7) for 15 min and incubated for 1 h at RT with Streptavidin‐HRP (Beyotime GS009‐6) at a 1:2000 dilution in the blocking buffer. Then, the membranes were washed four times with 1× washing buffer (Beyotime GS009‐8). Finally, the membranes were imaged using SuperSignal West Pico PLUS (Thermo 34 580) by GE AI680RGB.

## Conflict of Interest

The authors declare no conflict of interest.

## Author Contributions

Y.L., Z.L., and P.W. contributed equally to this work. L.M. and Z.L. conceived and supervised the project. Y.L. performed the experiments, with the help from P.W., Z.L. (Zhaohui Liang), Z.Y., and K.N. Z.L. conducted the data analysis. Y.L., L.M., and Z.L. wrote the manuscript with the help of the other authors. All authors read and approved the manuscript.

## Supporting information

Supporting Information

Supplemental Table 1

Supplemental Table 2

## Data Availability

The sequencing data generated in this study has been deposited in the NCBI GEO database under the accession number GSE235152.
