# Supplementary material for: The Transpeptidase Sortase A Binds Nucleic Acids and Mediates Mammalian Cell Labeling
Source: Adv Sci (Weinh). 2024 Apr 5;11(21):2305605. doi: 10.1002/advs.202305605 (PMC11151058; doi:10.1002/advs.202305605)
Supplement: Supplementary file 1 — Supporting Information [file ADVS-11-2305605-s002.pdf]

ADVANCED SCIENCE
Open Access

Supporting Information

for *Adv. Sci.*, DOI 10.1002/advs.202305605

The Transpeptidase Sortase A Binds Nucleic Acids and Mediates Mammalian Cell Labeling

*Yingzheng Liu, Zhike Lu, Panfeng Wu, Zhaohui Liang, Zhenxing Yu, Ke Ni and Lijia Ma**

Supporting Information

The transpeptidase sortase A binds nucleic acids and mediates mammalian cell labeling

*Yingzheng Liu, Zhike Lu, Panfeng Wu, Zhaohui Liang, Zhenxing Yu, Ke Ni, Lijia Ma**

Supporting Information

1. Supplementary Figures

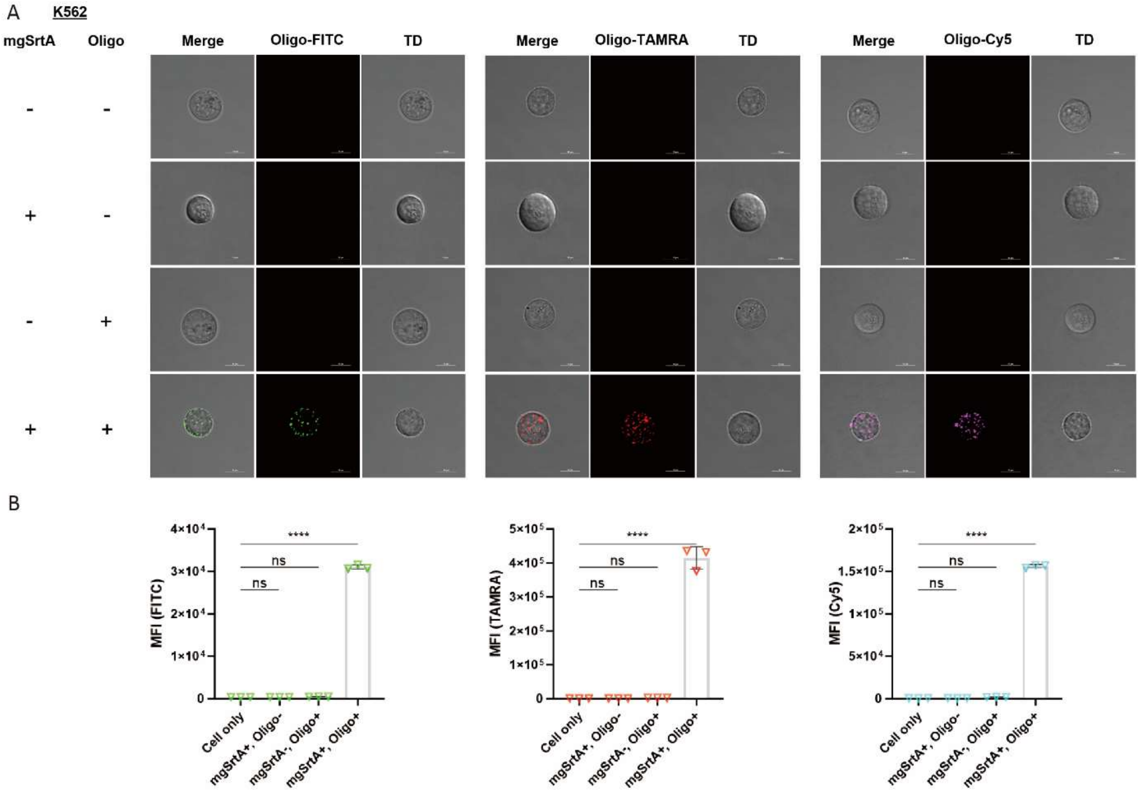

Figure S1. K562 labeled with oligonucleotides modified by FITC, TAMRA, Cy5 respectively. A) Images of labeled K562. Green, signal of FITC-modified oligonucleotide. Red, signal of TAMRA-modified oligonucleotide. Purple, signal of Cy5-modified oligonucleotide. Scale bar, 10 μm. B) Summary plot of K562 cells analyzed quantitatively by flow cytometry. Left, cells labeled by FITC modified oligonucleotide. Middle, cells labeled by TAMRA modified oligonucleotide. Right, cells labeled by Cy5 modified oligonucleotide. MFI, mean fluorescence intensity. The data represent mean ± SD. Experiments were conducted in triplicates. P-values are calculated using one-way ANOVA, **** $p < 0.0001$.

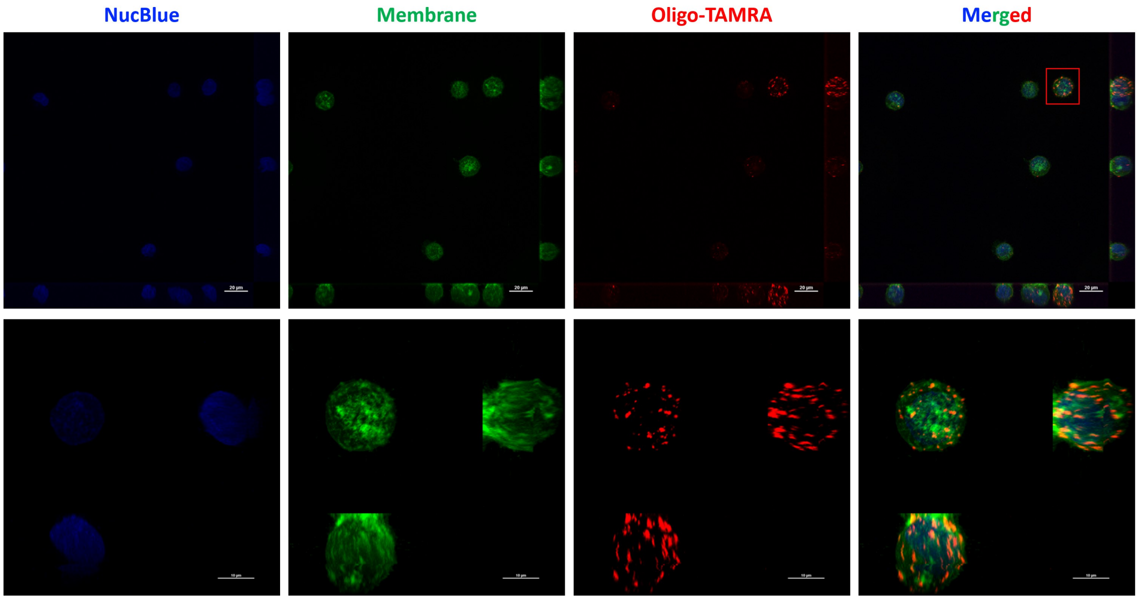

Figure S2. Orthogonal view of mgSrtA-mediated cell labeling. Nucleus: staining with NucBlue; Membrane: staining with CellMask Green; Oligonucleotide: visualized with via TAMRA modification. Cell in red box was zoomed. Scale bar: up, 20 μm; bottom, 10 μm.

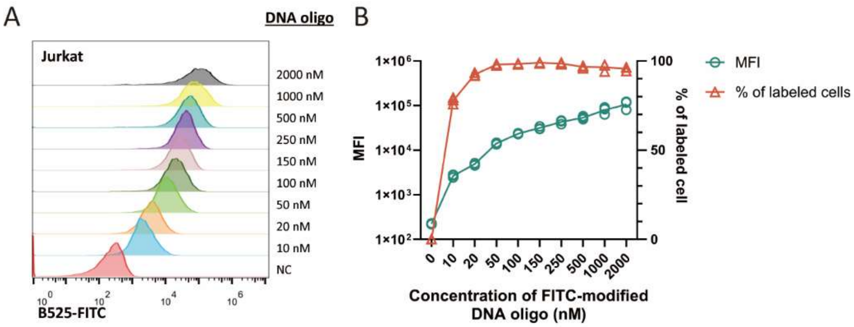

Figure S3. Concentration-dependent cell labeling. A) Flow cytometry quantification of the Jurkat cells labeled with FITC-modified DNA oligonucleotides. B) Summary plot of MFIs (mean fluorescence intensities) and percentages of Jurkat cells labeled with FITC-modified DNA oligonucleotides at different concentrations. Experiment was repeated 3 times with similar results.

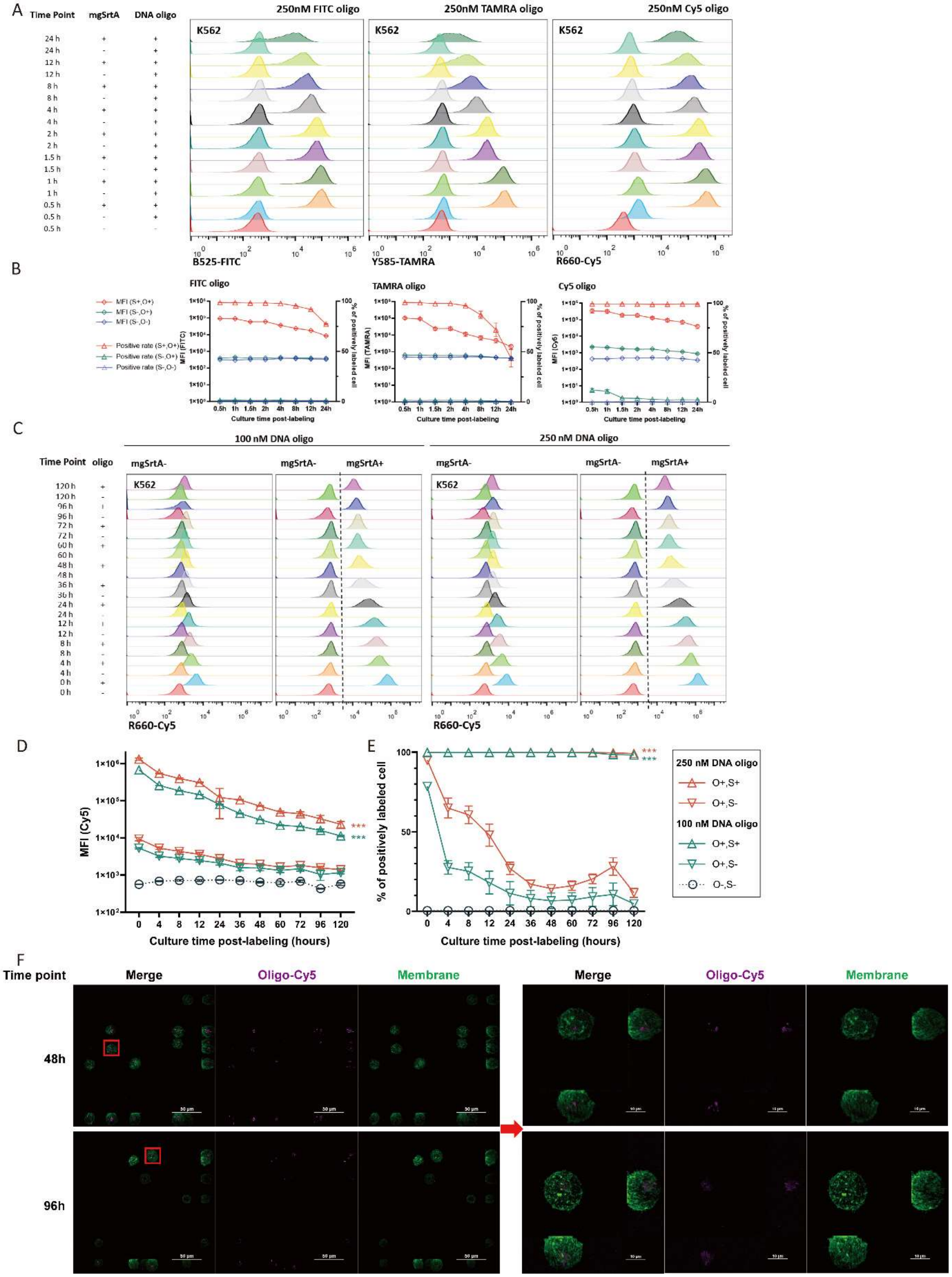

Figure S4. The fluorescence signals of the positively labeled cells were detectable 120 hours post labeling. A) The FITC, TAMRA, Cy5-modified DNA oligonucleotide was used to label cells, and the flourescent signals were quantified within 24 hours but in smaller time intervals. B) Summary plot of the MFI (mean fluorescence intensity) and the percentage of positively labeled cells at 24 hours. S: mgSrtA; O: DNA oligonucleotide. Data are represented as mean

± SD of three replicates. C) Cy5-modified DNA oligonucleotides (100 nM or 250 nM) were used to label K562 cells, and the signals were quantified by flow cytometry at multiple time points within 120 hours. After labeling, the cells were cultured under standard cell culture conditions over the course of the 120-hour experiments. D) Summary plot of the MFI (mean fluorescence intensity) over 120 hours. E) Summary plot of the percentage of positively labeled cells over 120 hours. Data are represented as mean ± SD of three replicates. F) Cy5-modified oligonucleotide internalized into the K562 cells after labeling. Cells in red box were zoomed. Green, membrane. Purple, Cy5-modified oligonucleotide. Scale bar: left, 50 μm; right, 10 μm. *** p< 0.001 by paired 2-tailed *t*-test.

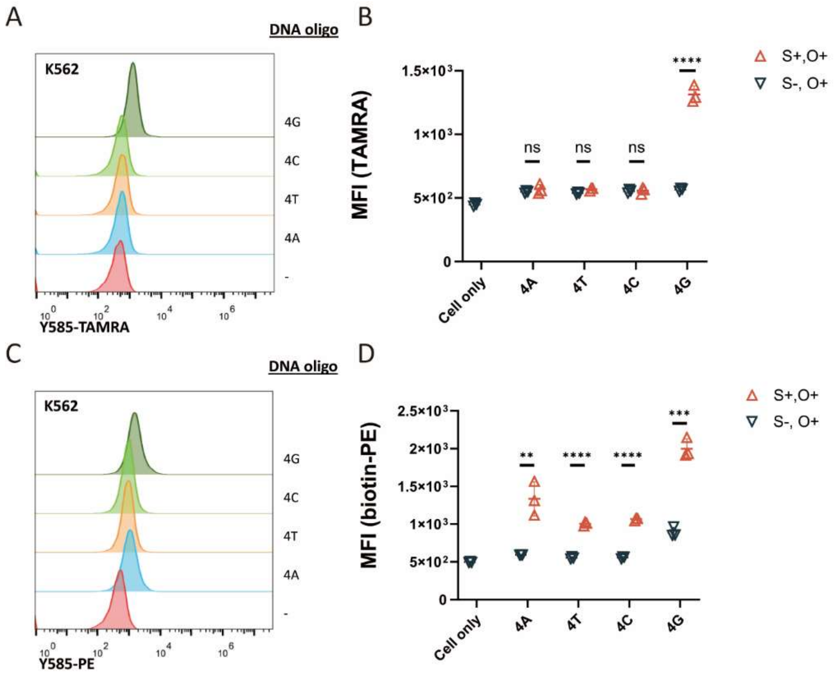

Figure S5. The signals of labeled TAMRA-modified (A and B) and biotin-modified (C and D) oligonucleotides on the cell surface were quantified by flow cytometry. S: mgSrtA; O: the 4-mer DNA oligonucleotides. Data are represented as mean ± SD of three replicates. ns: not significant, ** p < 0.01, *** p< 0.001, ****p < 0.0001 by unpaired 2-tailed *t*-test.

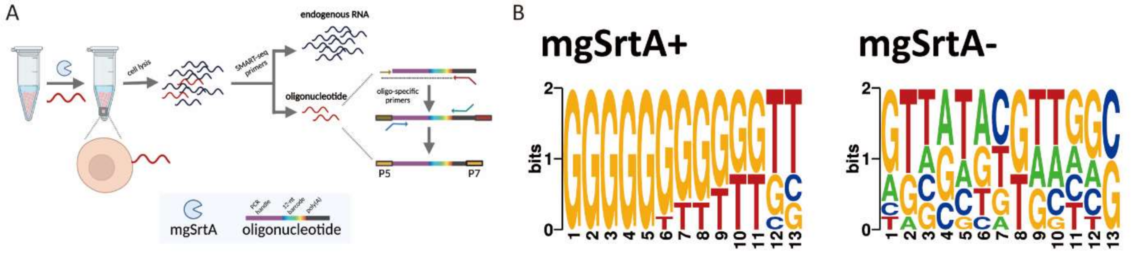

Figure S6. The sequence screening for preferred nucleotides in mgSrtA-mediated cell labeling. A) Schematic flowchart B) The weblogo of the screening.

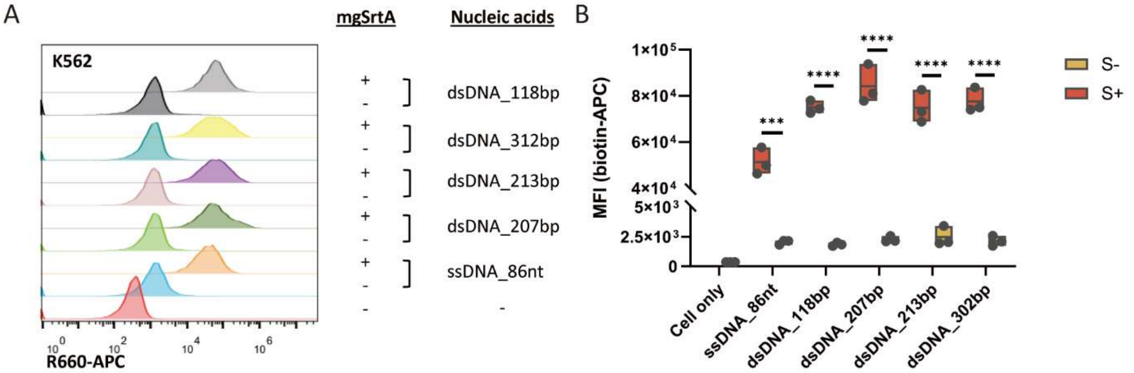

Figure S7. K562 cells labeled with dsDNA. A) Both double-stranded (ds) and single-stranded (ss) DNA were used in cell labeling mediated by mgSrtA. 0.5 million K562 cells were incubated with 20 μM mgSrtA at 37°C for 5 min and incubated for another 10 min in the presence of 40 nM oligonucleotides. Equimolar quantities of dsDNA and ssDNA were used in this quantification, in which each dsDNA molecule carried double the amount of biotin modification compared to ssDNA. B) Summary plot of K562 cells labeled with biotin-modified dsDNA and ssDNA. MFI, mean fluorescence intensity. S: mgSrtA. Experiment was conducted in triplicates. *** $p < 0.001$, **** $p < 0.0001$ by unpaired 2-tailed *t*-test.

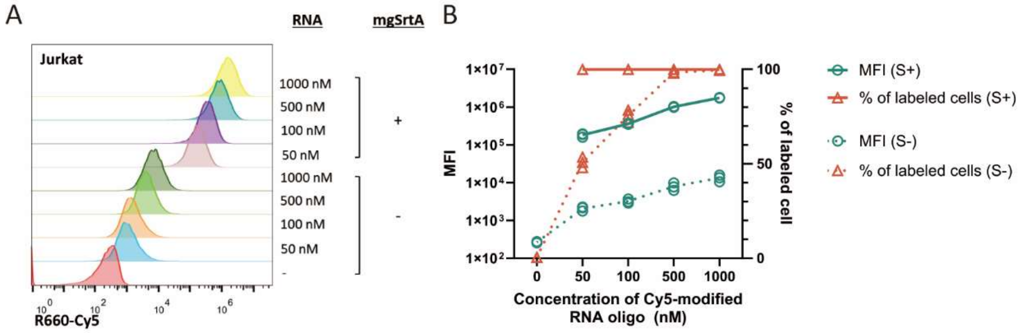

Figure S8. mgSrtA mediates Jurkat cell labeling with Cy5-modified RNA oligonucleotides. A) Flow cytometry quantification of Jurkat cells labeled with Cy5-modified RNA oligonucleotides. B) Summary plot of Jurkat cells labeled with Cy5-modified RNA oligonucleotides at different concentrations. MFI, mean fluorescence intensity. 0.5 million Jurkat cells were incubated with 20 μM mgSrtA at 37°C for 5 min and incubated for another 10 min in the presence of oligonucleotides. S: mgSrtA. Experiment was conducted in triplicates.

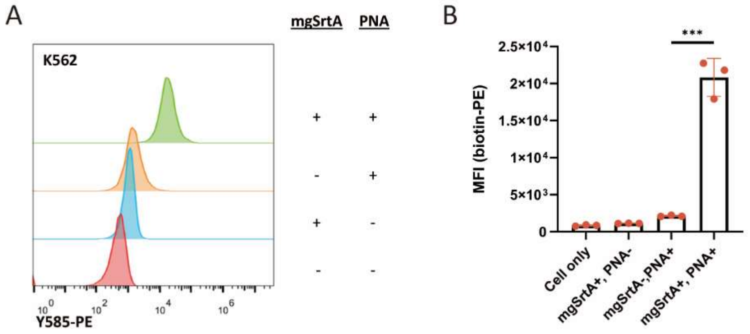

Figure S9. mgSrtA mediates K562 cell labeling with a biotin-modified peptide nucleic acid (PNA). A) Flow cytometry quantification of the K562 cells labeled with biotin-modified PNA. B) Summary plot of K562 cells labeled with biotin-modified PNA. MFI, mean fluorescence intensity. Data are represented as mean ± SD of three replicates. *** $p < 0.001$ by unpaired 2-tailed *t*-test. 0.5 million K562 cells were incubated with 20 μM mgSrtA at 37°C for 5 min and incubated for another 10 min in the presence of 500 nM PNA.

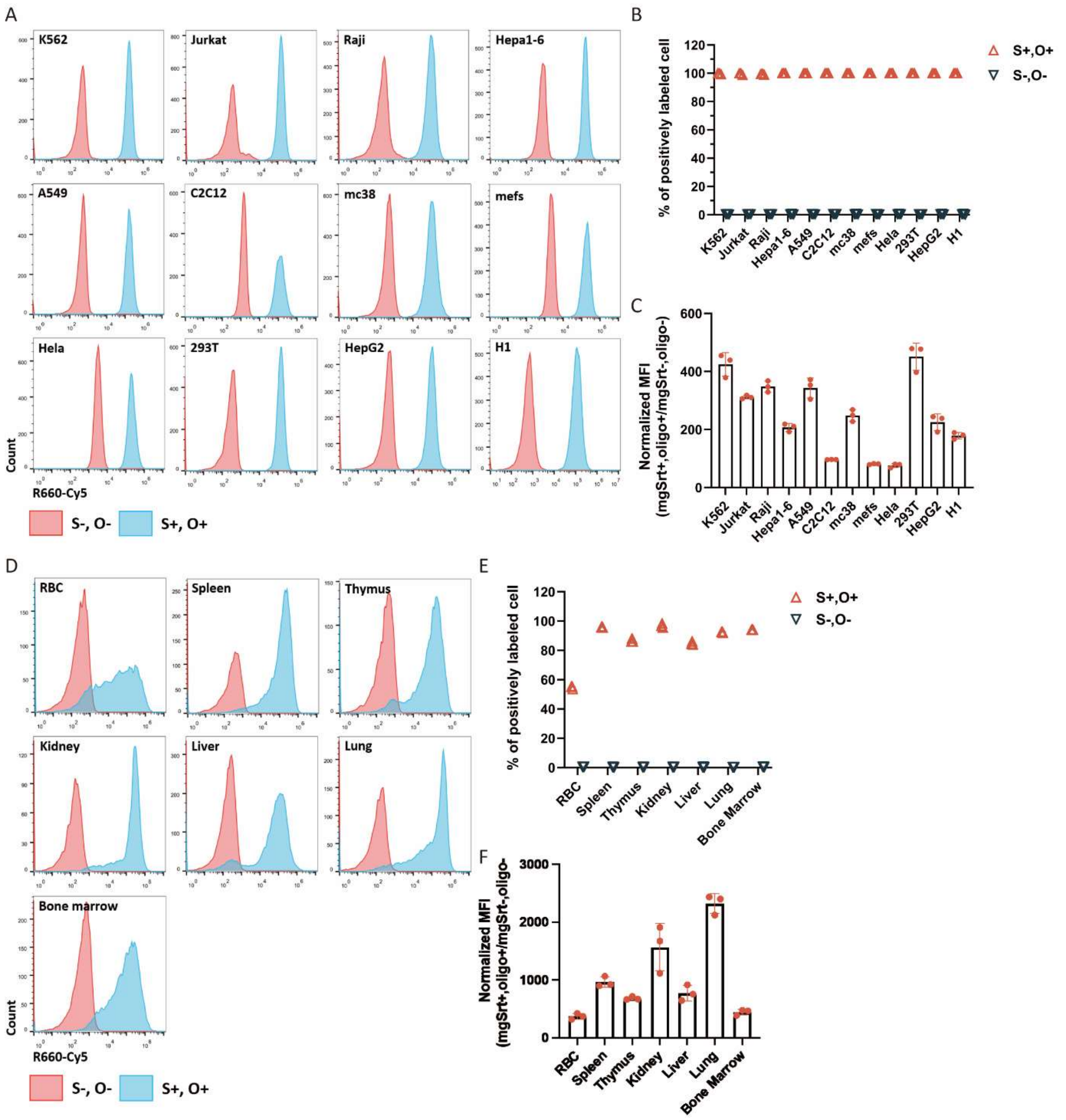

Figure S10. Cell labeling can be applied to a variety of cell lines. We applied the oligonucleotides to cells of multiple types in the presence of mgSrtA. A) Flow cytometry quantification of cultured cells of twelve different cell lines. B) Summary plot of the percentage of positively labeled cultured cells. Experiment was repeated 3 times with similar results. C) Summary plot of the normalized MFI (mean fluorescence intensity) of the cultured cells. Data are represented as mean ± SD of three replicates. D) Flow cytometry quantifications of cultured primary cells of seven different tissues. E) Summary plot of the percentage of positively labeled primary cells. Experiment was conducted in triplicates. F) Summary plot of the normalized MFI of the primary cells. Data are represented as mean ± SD of three replicates. S: mgSrtA; O: DNA oligonucleotide.

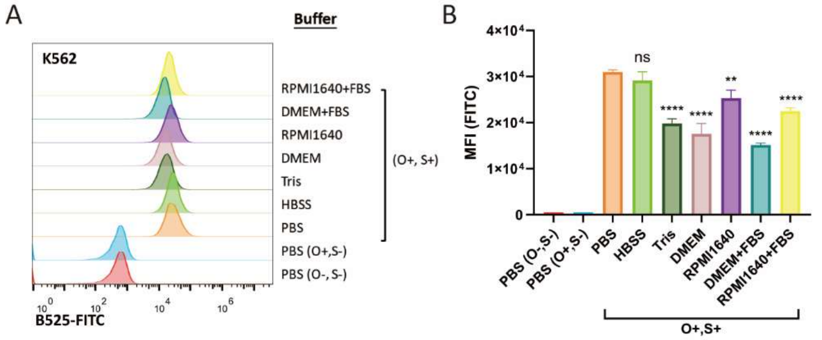

Figure S11. The efficiency of mgSrtA-mediated cell labeling measured in different buffers. A) K562 cells were incubated with mgSrtA and FITC-modified oligonucleotides in different buffers. B) Summary plot of K562 cells labeled by mgSrtA in different buffers. S: mgSrtA; O: FITC-modified oligonucleotides. MFI, mean fluorescence intensity. Data are represented as mean ± SD of three replicates. P-values are calculated using one-way ANOVA, ns: not significant, ** p < 0.01, **** p <0 .0001. PBS served as control.

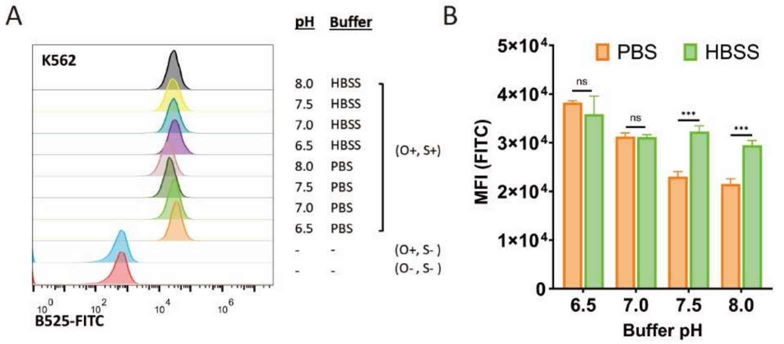

Figure S12. The efficiency of mgSrtA-mediated cell labeling measured at different pH values. A) K562 cells were incubated with mgSrtA and FITC-modified oligonucleotides at different pH values. B) Summary plot of K562 cells labeled by mgSrtA at different pH values. S: mgSrtA; O: FITC-modified oligonucleotides. MFI, mean fluorescence intensity. Data are represented as mean ± SD of three replicates. ns: not significant, *** p< 0.001 by unpaired 2-tailed *t*-test.

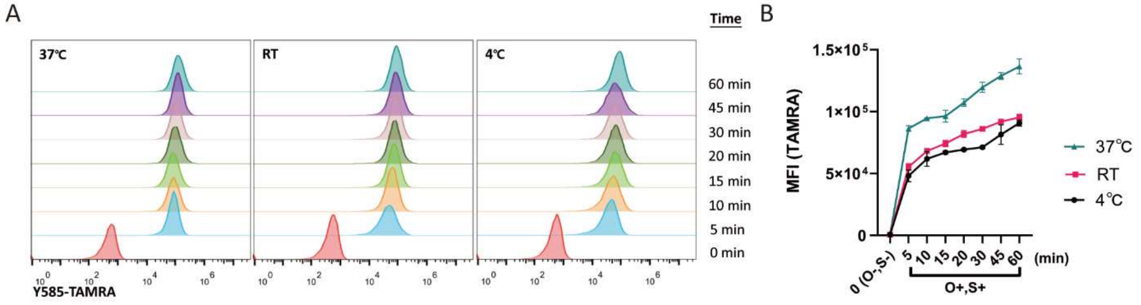

Figure S13. The efficiency of mgSrtA-mediated cell labeling measured at different temperatures. A) K562 cells were incubated with mgSrtA and TAMRA-modified oligonucleotides at 4°C or RT or 37°C. B) Summary plot of K562 cells labeled by mgSrtA at different temperatures. MFI, mean fluorescence intensity. Data are represented as mean ± SD of three replicates.

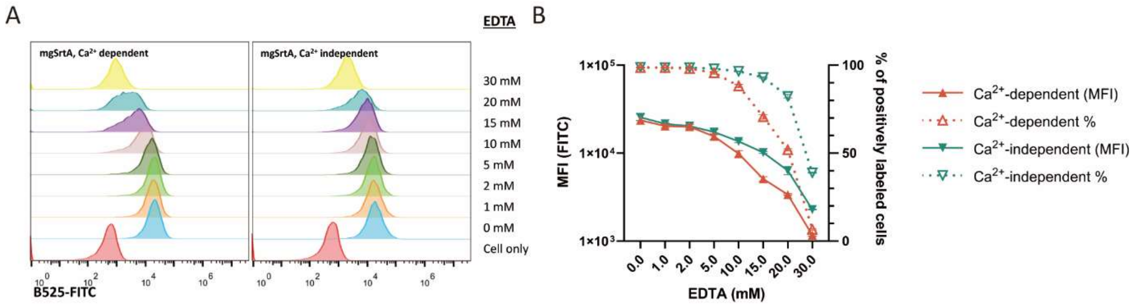

Figure S14. Quenching the labeling reaction by EDTA. A) K562 cells were incubated with mgSrtA and FITC-modified oligonucleotides in the presence of EDTA. B) Summary plot of mgSrtA-mediated cell labeling terminated at different EDTA concentrations. MFI, mean fluorescence intensity. Data are represented as mean ± SD of three replicates.

CellID	Cell type	Species
CA11	Hela	human
CA12	C2C12	mouse
CA13	293T	human
CA14	K562	human
CA15	A549	human
CA16	Hepa1-6	mouse
CA17	Jurkat	human
CA18	mc38	mouse

Figure S15. List of CellIDs, labeled cell types, and originating species used in multiplexed scRNA-seq.

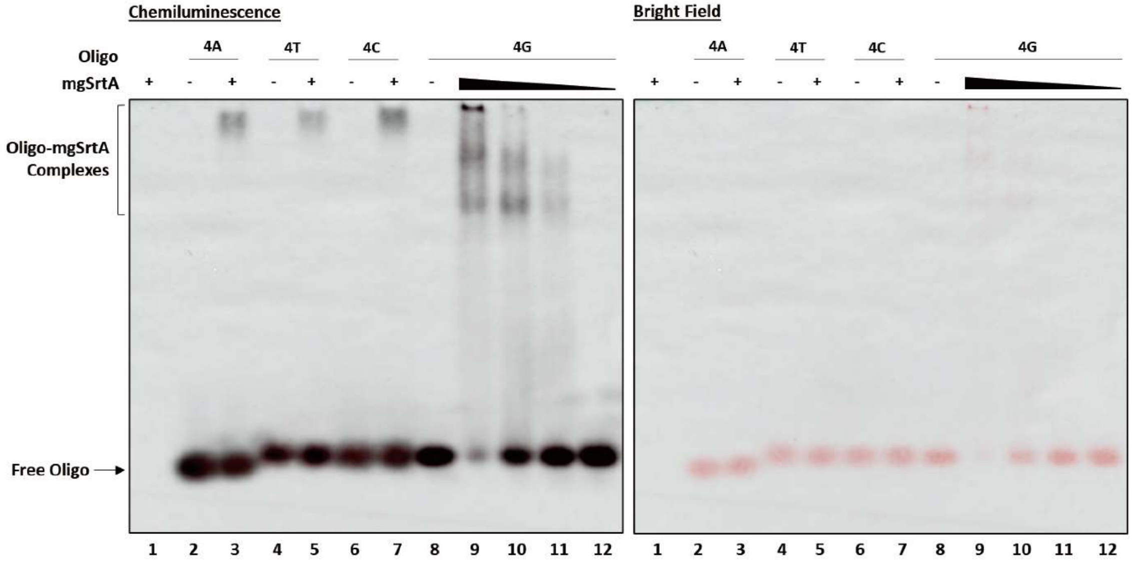

Figure S16. The EMSA of the binding between mgSrtA and 4-mer DNA oligonucleotides. 20 μM biotin-modified and FITC-modified oligonucleotides were incubate with 20 μM mgSrtA in lane 3, lane 5, lane 7 and lane 9. Lane 10 contains 10 μM mgSrtA, lane 11 contains 5 μM mgSrtA, lane 12 contains 1 μM mgSrtA. Left, chemiluminescence; right, bright field. The membrane was incubated with streptavidin-HRP.

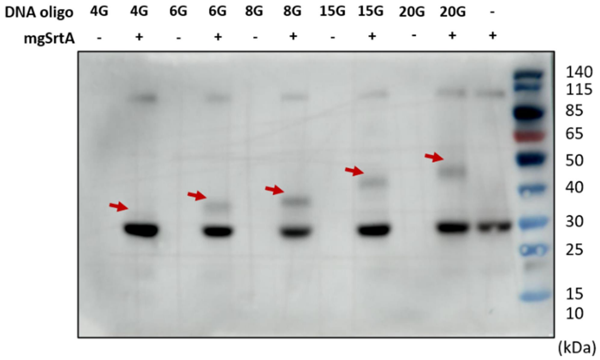

Figure S17. mgSrtA incubated with the oligonucleotides with increased length. (the 4G, 6G, 8G, 15G, and 20G oligonucleotides were biotinylated at the 5’ end). The red arrow points to the product bands. The membrane was incubated with a mouse anti-biotin primary antibody and a goat anti-mouse secondary antibody.

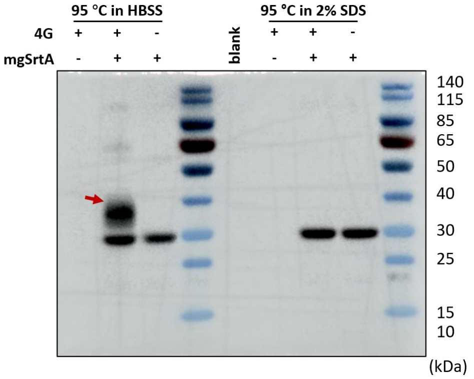

Figure S18. mgSrtA was pretreated with 2% SDS before incubated with the 4G DNA oligonucleotide (the DNA oligonucleotide was modfied by 5' TAMRA and 3' biotin). The red arrow points to the product band. The membrane was incubated with a mouse anti-biotin primary antibody and a goat anti-mouse secondary antibody.

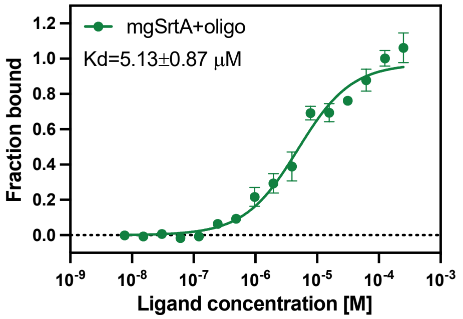

Figure S19. MST assays for the quantitation of the affinity of binding between mgSrtA and Cy5-modified oligonucleotides. Error bars represent the mean ± SEM based on four replicates.

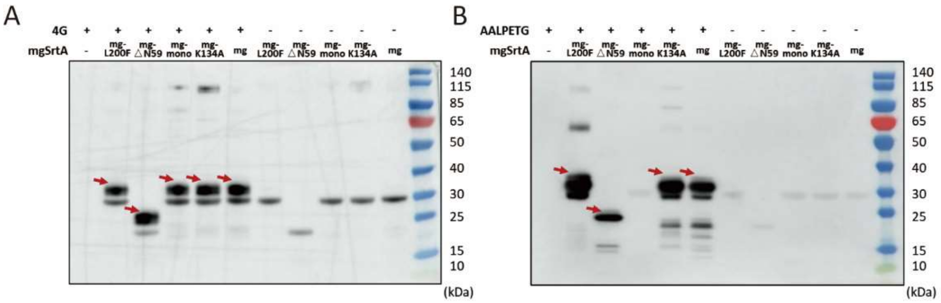

Figure S20. 10 μM mgSrtA mutants incubated with 20 μM FITC and biotin-modified 4G DNA oligonucleotide (A) or biotin-modified AALPETG peptide (B) at 37°C for 30 min. mgSrtA-mono represents the mgSrtA mutant with N132A, K137A, and Y143A mutations. The red arrow points to the product bands. The membranes were incubated with a mouse anti-biotin primary antibody and a goat anti-mouse secondary antibody.

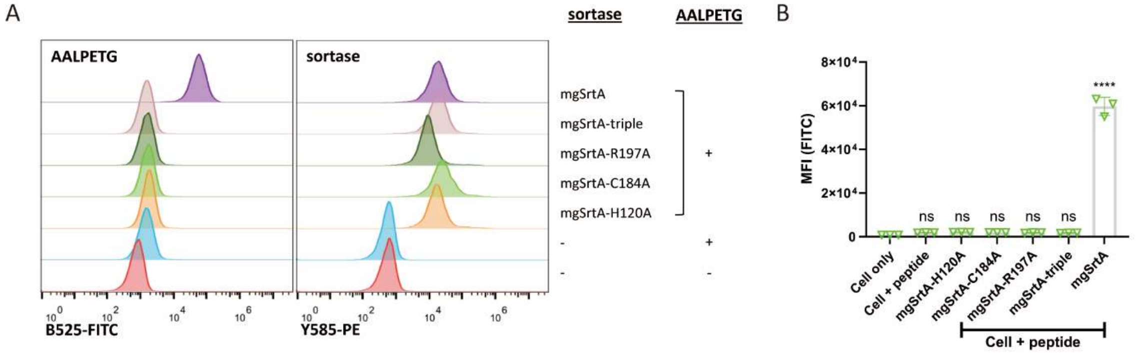

Figure S21. K562 cells were incubated with FITC-modified AALPETG peptide and mgSrtA mutants carrying the mutations H120A, C184A, or R197A and mgSrtA-triple. A) The signals of the labeled peptide and anchored mgSrtA or its mutants on cells were detected by flow cytometry. B) Summary plot of the signal of labeled FITC-modified peptide on K562 cells. MFI, mean fluorescence intensity. Data are represented as mean ± SD of three replicates. P-values are calculated using one-way ANOVA, ns: not significant, **** p <0 .0001.

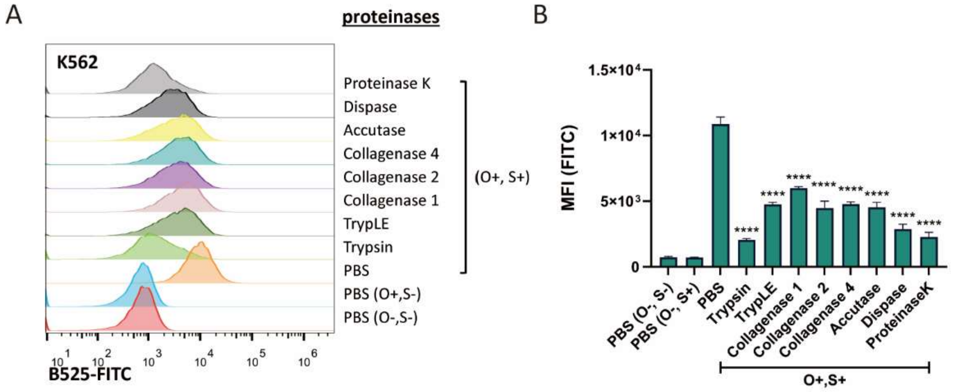

Figure S22. mgSrtA-mediated cell labeling conducted on K562 cells that were pretreated with various proteinases. A) K562 cells were incubated with various proteinases st 37°C for 5 min before labeled with FITC-modified oligonucleotides. B) The signal of labeled oligonucleotides on cells. S: mgSrtA; O: FITC-modified oligonucleotides. MFI, mean fluorescence intensity. Data are represented as mean ± SD of three replicates. P-values are calculated using one-way ANOVA, ns: not significant, **** p <0 .0001. PBS served as control.

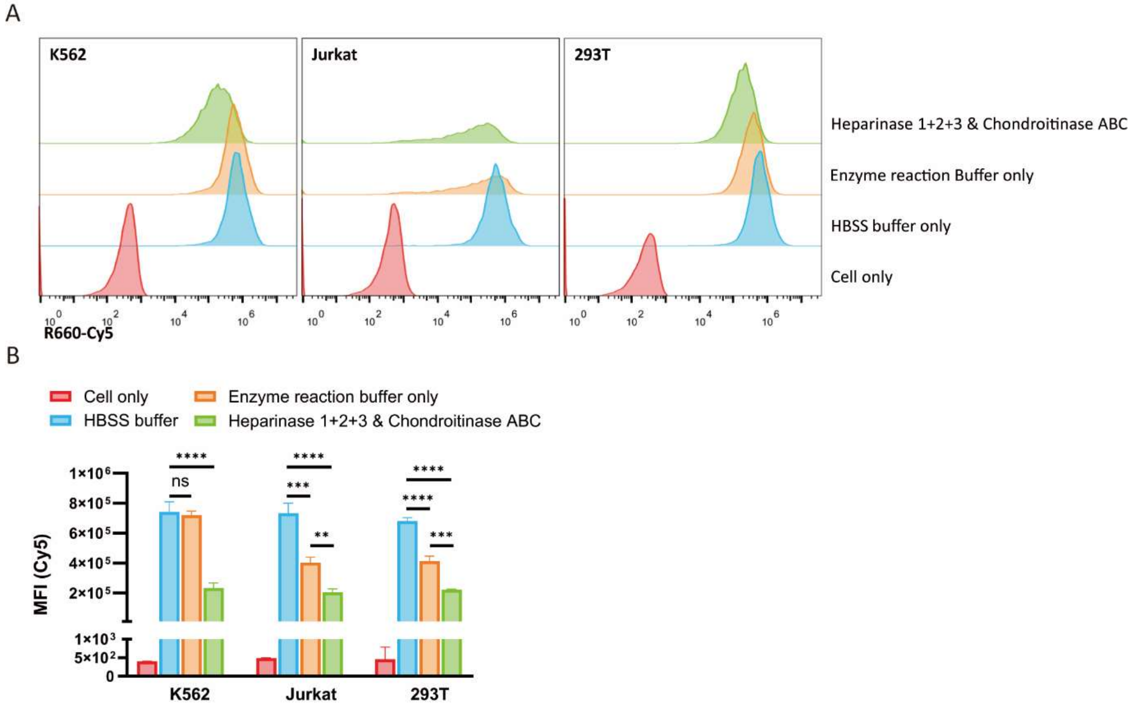

Figure S23. mgSrtA-mediated cell labeling conducted on K562, Jurkat, and 293T cells that were pretreated with heparinases and chondroitinases. A) The K562, Jurkat, and 293T cells were pretreated with heparinases and chondroitinases before labeled with Cy5-modified oligonucleotides. B) The signal of the labeled oligonucleotides on K562, Jurkat, and 293T cells. MFI, mean fluorescence intensity. Data are represented as mean ± SD of three replicates. P-values are calculated using one-way ANOVA, ns: not significant, ** $p < 0.01$, *** $p < 0.001$, **** $p < 0.0001$. Cells were pretreated with 120 U/mL Heparinase I, 40 U/mL Heparinase II, 7 U/mL Heparinase III and 1 U/mL Chondroitinase with 1× Heparinase reaction buffer in HBSS at 37°C for 1 h.

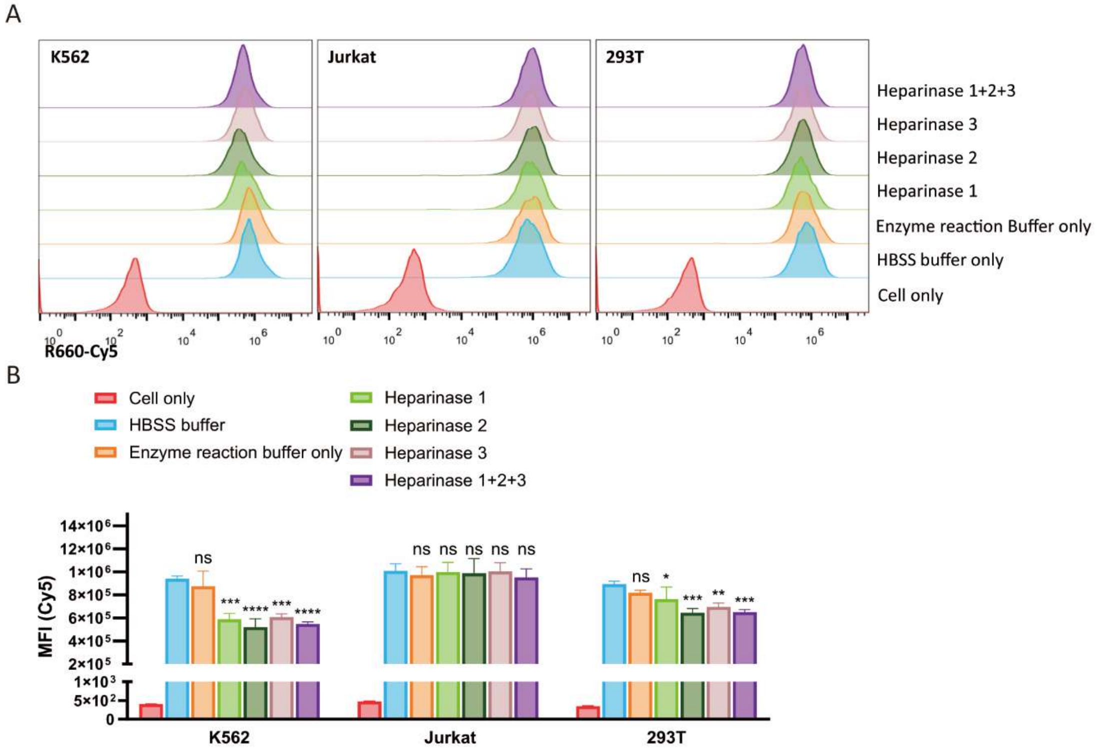

Figure S24. mgSrtA-mediated cell labeling conducted on K562, Jurkat, and 293T cells that were pretreated with heparinases. A) The K562, Jurkat, and 293T cells were pretreated with heparinases before labeled with Cy5-modified oligonucleotides. B) The signal of the labeled oligonucleotides on K562, Jurkat, and 293T cells. MFI, mean fluorescence intensity. Data are represented as mean ± SD of three replicates. P-values are calculated using one-way ANOVA, ns: not significant, * $p < 0.05$, ** p <0 .01, *** p <0 .001, **** p <0 .0001. HBSS buffer served as control. Cells were pretreated with 120 U/mL Heparinase I, 40 U/mL Heparinase II, 7 U/mL Heparinase III and the mix of three kinds of heparinases respectively with 1× Heparinase reaction buffer in HBSS at 30°C for 1 h.

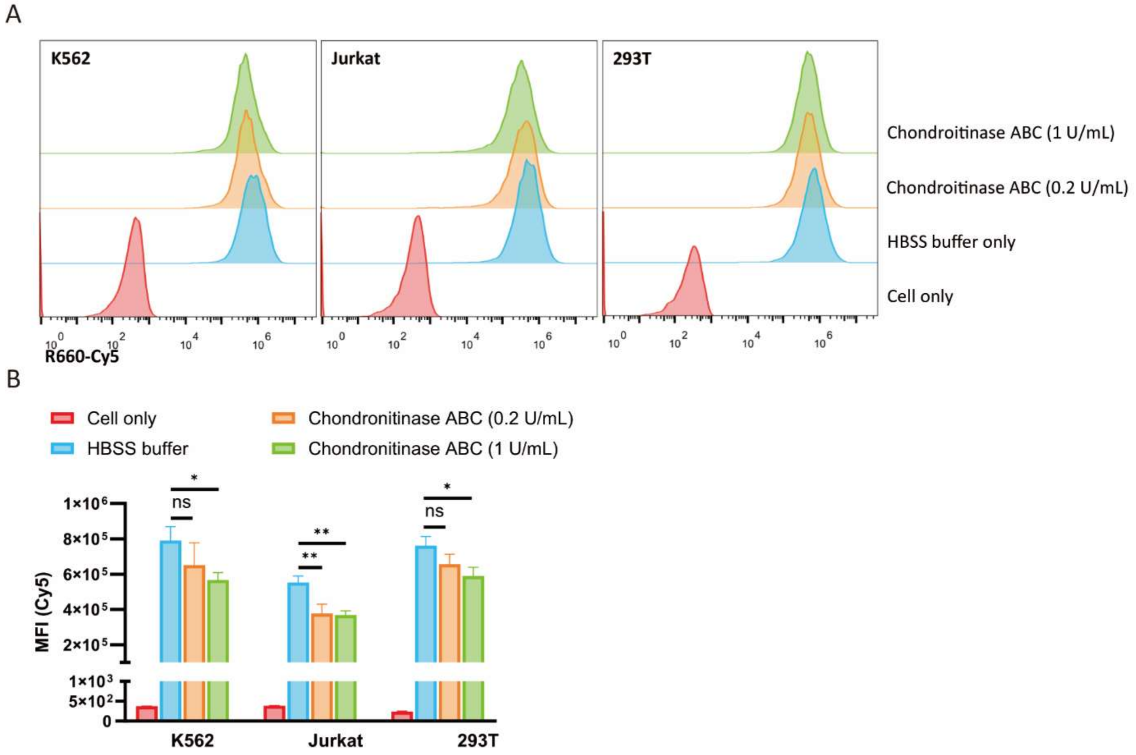

Figure S25. mgSrtA-mediated cell labeling conducted on K562, Jurkat, and 293T cells that were pretreated with chondroitinases. A) The K562, Jurkat, and 293T cells were pretreated with chondroitinases before labeled with Cy5-modified oligonucleotides. B) The signal of the labeled oligonucleotides on K562, Jurkat, and 293T cells. MFI, mean fluorescence intensity. Data are represented as mean ± SD of three replicates. P-values are calculated using one-way ANOVA, ns: not significant, * $p < 0.05$, ** $p <0 .01$. Cells were pretreated with 0.2 U/mL or 1 U/mL Chondroitinase in HBSS at 37°C for 1 h.

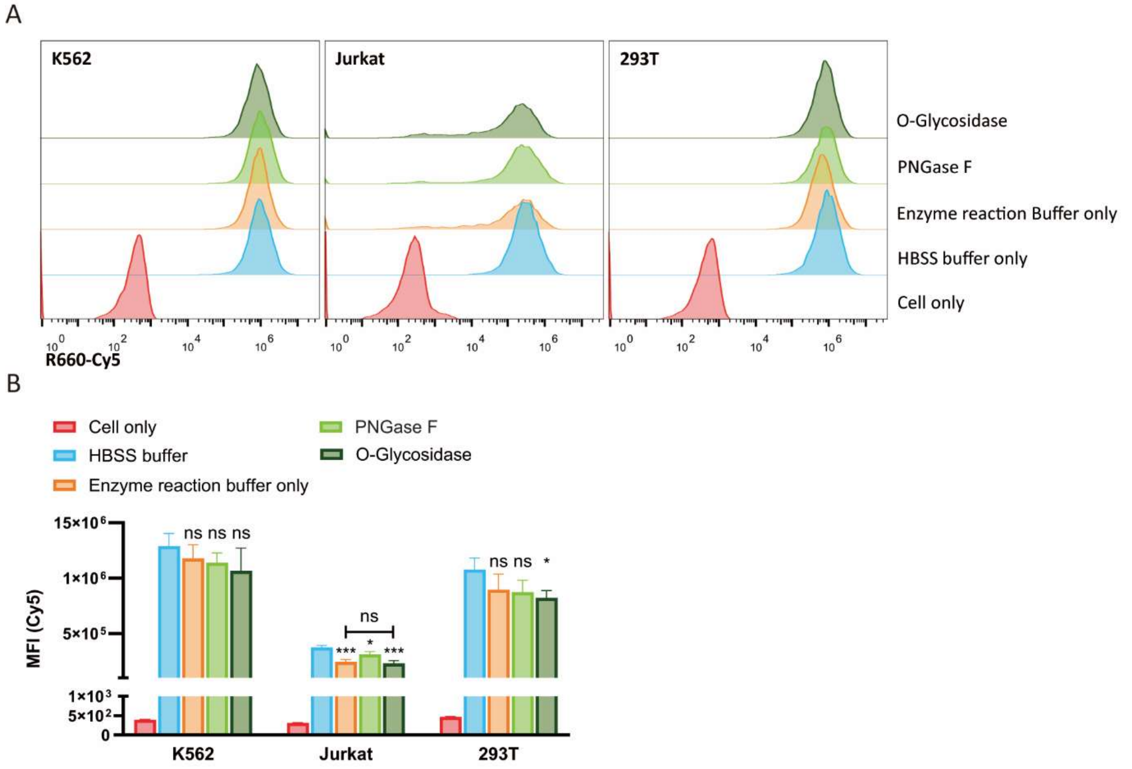

Figure S26. mgSrtA-mediated cell labeling conducted on K562, Jurkat, and 293T cells that were pretreated with O-glycosidase or PNGase F. A) The K562, Jurkat, and 293T cells were pretreated with O-glycosidase or PNGase F before labeled with Cy5-modified oligonucleotides. B) The signal of the labeled oligonucleotides on K562, Jurkat, and 293T cells. MFI, mean fluorescence intensity. Data are represented as mean ± SD of three replicates. P-values are calculated using one-way ANOVA, ns: not significant, * $p < 0.05$, *** $p < 0.001$. HBSS buffer served as control. Cells were treated with 40 kU/mL O-Glycosidase or 5 kU/mL PNGase F respectively with 1× GlycoBuffer 2 in HBSS at 37 °C for 1 h.

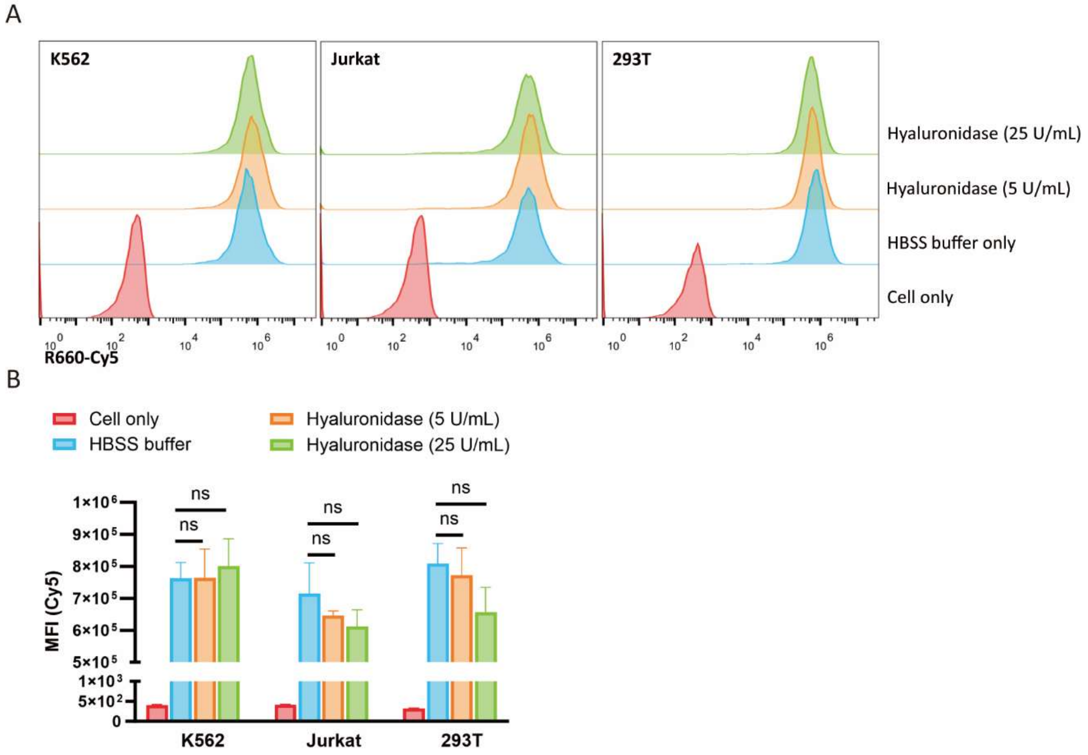

Figure S27. mgSrtA-mediated cell labeling conducted on K562, Jurkat, and 293T cells that were pretreated with hyaluronidases. A) The K562, Jurkat, and 293T cells were pretreated with hyaluronidases before labeled with Cy5-modified oligonucleotides. B) The signal of the labeled oligonucleotides on K562, Jurkat, and 293T cells. MFI, mean fluorescence intensity. Data are represented as mean ± SD of three replicates. P-values are calculated using one-way ANOVA, ns: not significant. Cells were pretreated with 5 U/mL or 25 U/mL Hyaluronidase in HBSS at 37°C for 1 h.

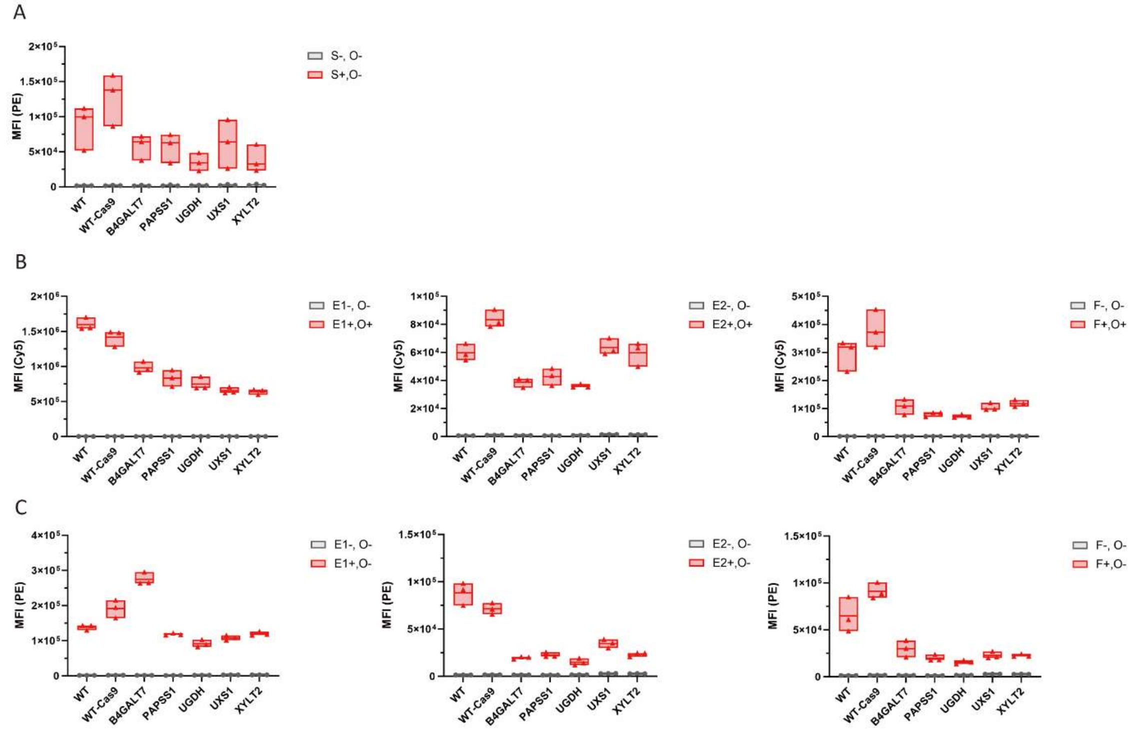

Figure S28. Validation of the CRISPR screening candidates. Candidate genes were knocked out from K562 cells to establish the engineered K562 cells. A) K562 knocking out cell lines were incubated with mgSrtA. S: mgSrtA; O: oligonucleotide. B) K562 knocking out cell lines were labeled with Cy5-modified oligonucleotides mediated by SrtE1, SrtE2, SrtF, respectively. C) K562 knocking out cell lines were incubated with SrtE1, SrtE2, SrtF, respectively. E1: SrtE1; E2: SrtE2; F: SrtF. MFI, mean fluorescence intensity. Experiment was conducted in triplicates.

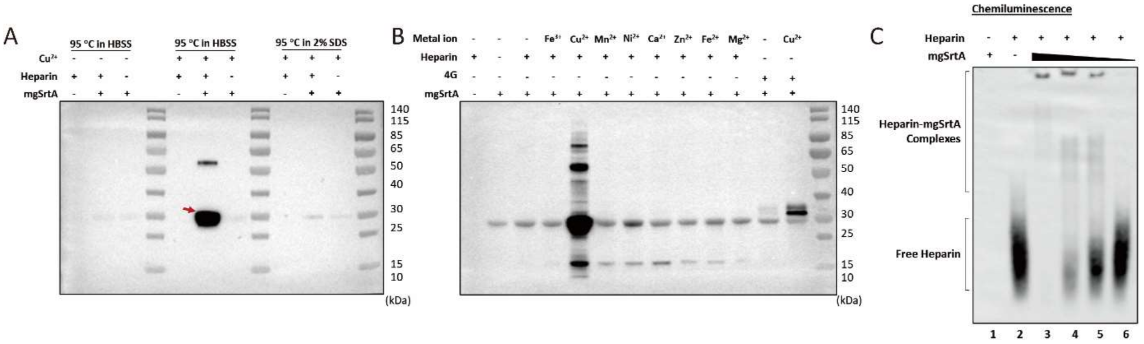

Figure S29. The reaction between mgSrtA and heparin *in vitro*. A) 10 μM mgSrtA incubated with 300 ng/μL biotin-modified heparin at 37°C for 30 min in the presence of 100 μM Cu^{2+}. mgSrtA was pretreated in 2% SDS at 95 °C for 10 min and returned to room temperature before incubated with heparin.The red arrow points to the product bands. B) mgSrtA

incubated with biotin-modified heparin in the presence of metal ions. Western Blots were incubated with a mouse anti-biotin primary antibody and a goat anti-mouse secondary antibody. C) The heparin-mgSrtA complexes in EMSA. 100 ng/μL heparin was incubated with 20 μM, 10 μM, 5 μM, 1 μM mgSrtA respectively. The EMSA was incubated with streptavidin-HRP.

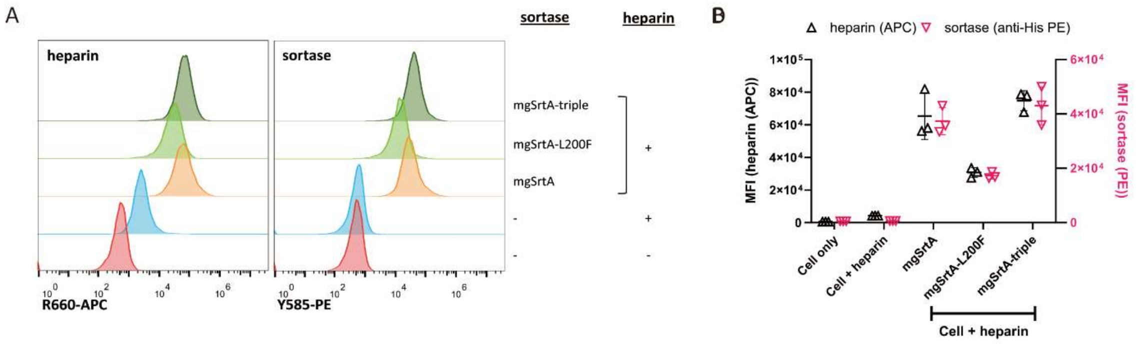

Figure S30. K562 cells were labeled with biotin-modified heparin mediated by mgSrtA, mgSrtA-L200F, and mgSrtA-triple. mgSrtA-triple represents the mgSrtA mutant with H120A, C184A, and R197A mutations. A) Flow cytometry quantification of the labeled K562. B) The signal of the labeled heparin on K562 ceslls. MFI, mean fluorescence intensity. Data are represented as mean ± SD of three replicates. 0.5 million K562 cells were incubated with 20 μM mgSrtA at 37°C for 5 min and incubated for another 10 min in the presence of 3 ng/μL heparin.

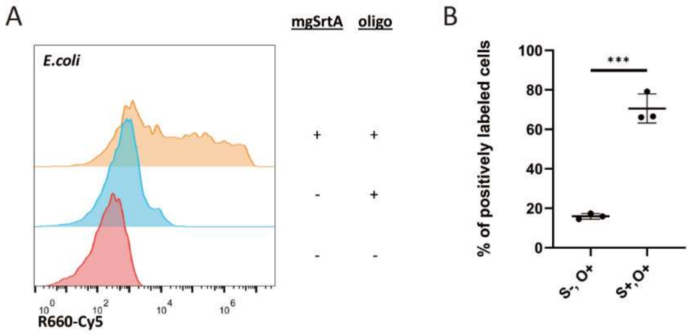

Figure S31. mgSrtA mediates *E.coli* labeling with oligonucleotides. A) Flow cytometry quantification of the *E.coli* labeled with Cy5-modified oligonucleotides. B) The percentage of positively labeled *E.coli*. S: mgSrtA; O: oligonucleotide. Data are represented as mean ± SD of three replicates. *** p< 0.001 by unpaired 2-tailed *t*-test. 5 millions *E.coli* were incubated

with 20 µM mgSrtA at 37°C for 5 min and incubated for another 10 min in the presence of 1 µM Cy5-modified oligonucleotides.

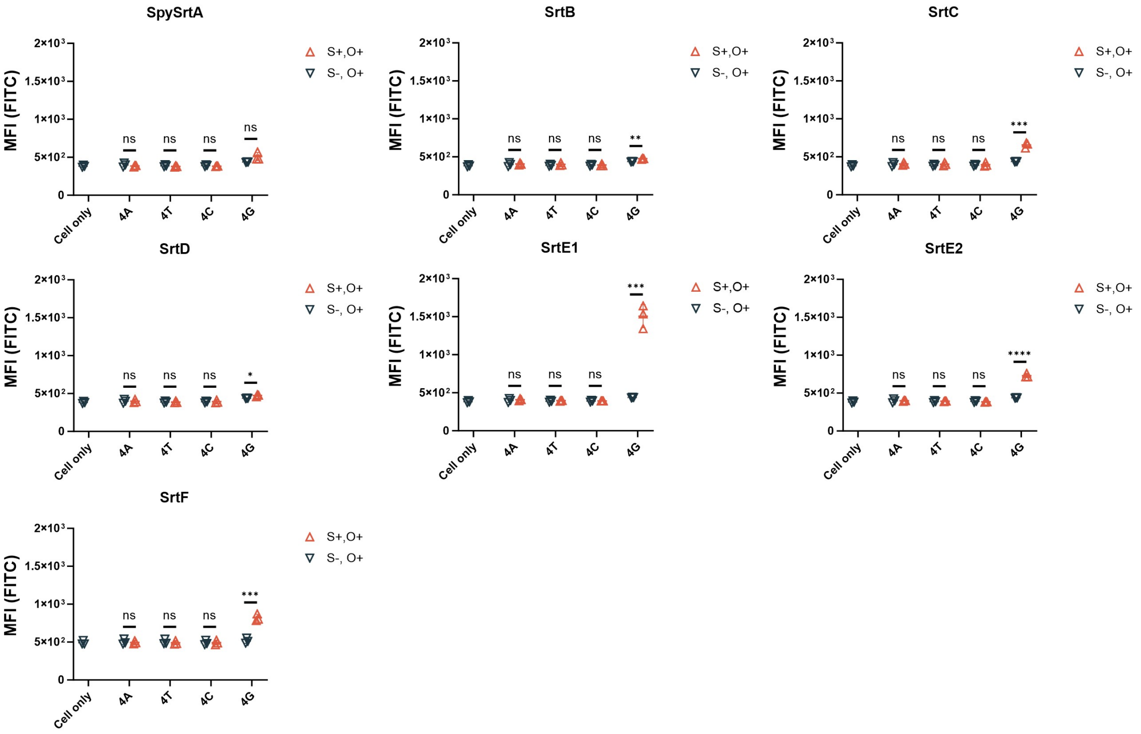

Figure S32. Various wild-type sortases were used to label the surface of K562 cells with FITC-modified 4-mer oligonucleotides (A: 4A oligonucleotide; T: 4T oligonucleotide; C: 4C oligonucleotide; G: 4G oligonucleotide). S: mgSrtA; O: oligonucleotide. Data are represented as mean ± SD of three replicates. ns: not significant, * $p < 0.05$, ** $p < 0.01$, *** $p < 0.001$, **** $p < 0.0001$ by unpaired 2-tailed *t*-test..

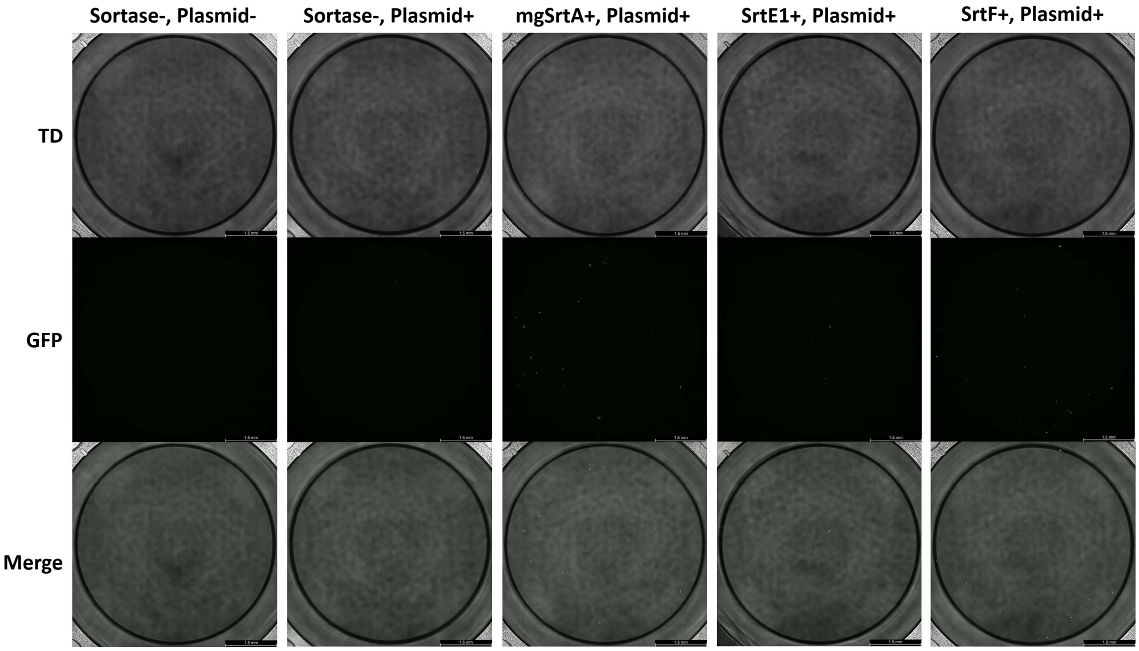

Figure S33. The picture of HEK-293T in 48-well plate 72 h after labeled with GFP-expressing plasmid. Scale bar, 1.5 mm. Experiment was repeated 3 times with similar results.

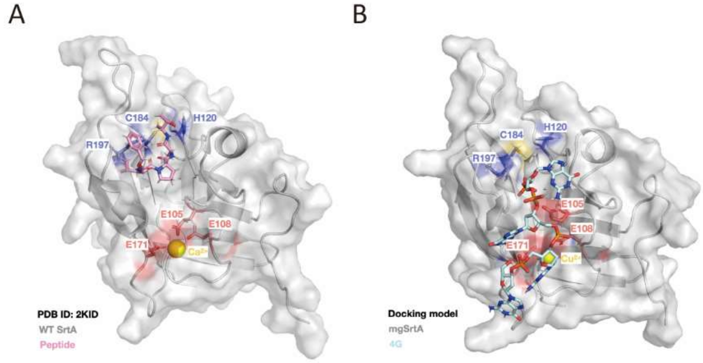

Figure S34. The resultant docking model. A) Reported crystal structure of wild-type sortase A and peptide. B) Docking simulation of the 4G DNA oligonucleotide and mgSrtA.

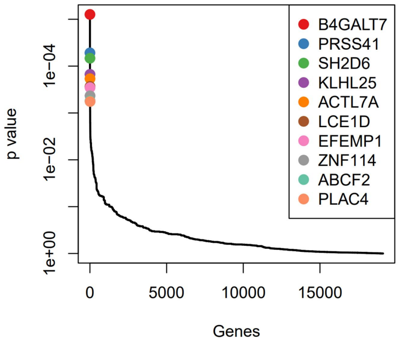

Figure S35. The top hits of CRISPR screening of genes that facilitate LPETG cell labeling. Genes are ranked (x-axis) by p value (y-axis).

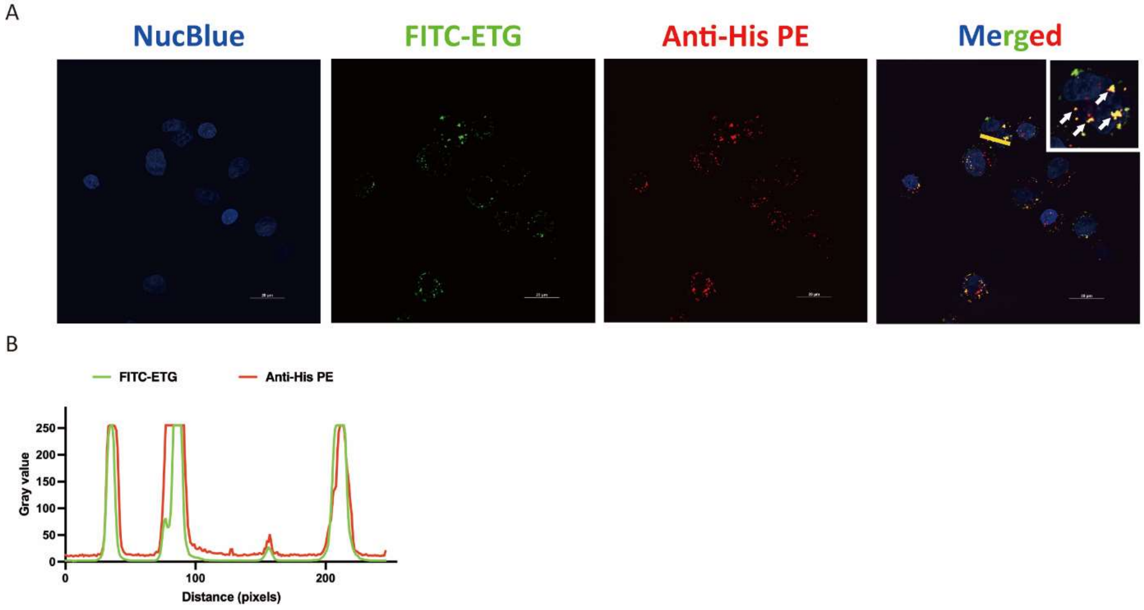

Figure S36. The colocalization of the labeled LPETG peptide and anchored mgSrtA observed by confocal microscopy. A) Confocal images of K562 cells labeled with FITC-modified LPETG peptide and incubated with anti-his PE antibody. Blue, nucleus. Green, peptide. Red, mgSrtA. Yellow, the colocalization of mgSrtA and the oligonucleotide. The white arrow points to the colocalization sites. Scale bar, 20 μm. B) Fluorescence intensity profiles along the yellow line in the merged image.

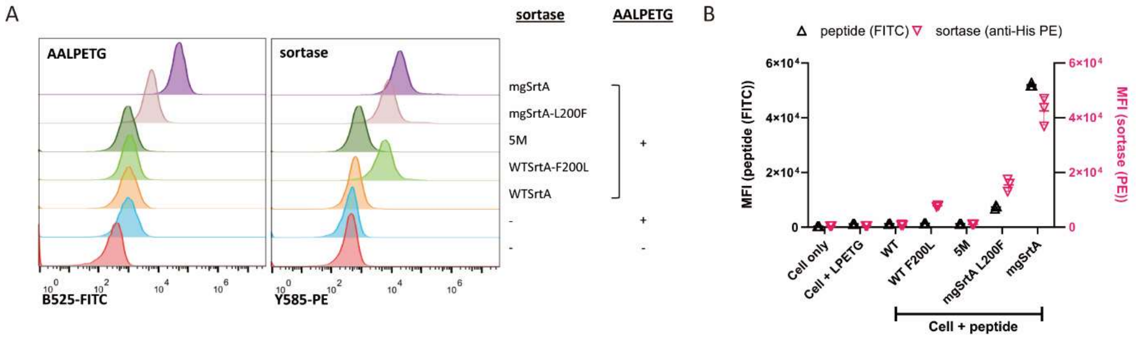

Figure S37. K562 cells were labeled with FITC-modified LPETG peptide. A) The signals of the labeled oligonucleotide and anchored WTSrtA or its mutants on cells were detected by flow cytometry. B) Summary plot of K562 cells labeled with FITC-modified LPETG peptide mediated by WTSrtA and its mutants. Data are represented as mean ± SD of three replicates.

2. Supplementary Tables

Tabel S1. The sequences of substrates and reaction conditions.

Tabel S2. The sequences of sortases.
